# In vivo treatment of tyrosinaemia with hypercompact Cas12f1

**DOI:** 10.1038/s41421-023-00554-y

**Published:** 2023-07-18

**Authors:** Ruochen Guo, Zhifang Li, Guoling Li, Hainan Zhang, Chang Zhang, Xiaona Huo, Xiaoyin Zhang, Xiali Yang, Rongrong Yang, Yuanhua Liu, Xiaozhi Sun, Xinyu Liu, Hui Yang, Yingsi Zhou, Chunlong Xu

**Affiliations:** 1 Lingang Laboratory, Shanghai, China; 2 Institute of Neuroscience, State Key Laboratory of Neuroscience, Key Laboratory of Primate Neurobiology, CAS Center for Excellence in Brain Science and Intelligence Technology, Shanghai Institutes for Biological Sciences, Chinese Academy of Sciences, Shanghai, China; 3 Shanghai Center for Brain Science and Brain-Inspired Technology, Shanghai, China; 4 HuidaGene Therapeutics Inc, Shanghai, China; 5 School of Life Sciences and Technology, ShanghaiTech University, Shanghai, China

**Keywords:** Biological techniques, Cell biology

Dear Editor,

Different subtypes of CRISPR associated Cas12 proteins exhibit extensive diversity^1^. In contrast to Cas12a and Cas12b, Cas12f1 is hypercompact in size with only 400 to 500 amino acids (a.a.) residues, and is lately demonstrated as active nuclease in cultured cells^2–6^. However, in vivo activity and therapeutic potential of Cas12f1 remain to be investigated. Taking advantage of its miniature size for in vivo delivery with an all-in-one single adeno-associated virus (AAV) vector, we herein showed that Cas12f1 enabled efficient in vivo editing in both mouse embryos and liver with minimal off-target effect. Moreover, targeted delivery of Cas12f1 by single AAV to liver rescued tyrosinaemia in mouse via inactivation of *Hpd* gene, indicating a great potential of Cas12f1 for treating genetic diseases.

Three different Cas12f1 orthologs were recently shown to possess nuclease activity in HEK293T cells^2–5^. To investigate their activity in mouse cells, we first designed 25 guide RNAs (gRNAs) for AsCas12f1, SpCas12f1 and Un1Cas12f1 targeting several genomic sites of mouse *Trp53* gene. Mouse N2a cells were transfected with three Cas12f1 orthologs and *Trp53*-targeting gRNAs (Fig. 1a), and gene editing efficiency was analyzed by deep-sequencing. Un1Cas12f1 yielded 10% gene editing rate in 5 out of 25 target sites; however, we observed no detectable indel by either AsCas12f1 or SpCas12f1 (Fig. 1b; Supplementary Table S1). Therefore, Un1Cas12f1 was chosen for the following experiments.

**Fig. 1:**
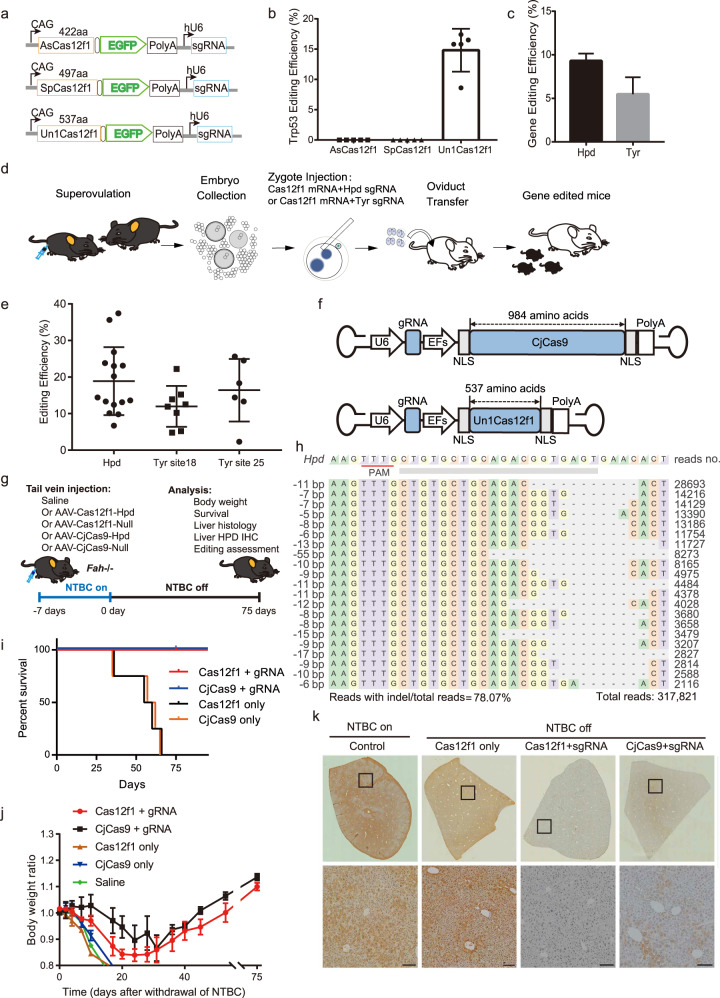
In vivo gene editing and disease treatment using hypercompact Un1Cas12f1 in a mouse model of tyrosinaemia. **a** Schematic map of three Cas12f1 orthologs (AsCas12f1, SpCas12f1 and Un1Cas12f1). **b** In vitro editing efficiency of three Cas12f1 orthologs for target sites of mouse *Trp53* gene in N2a cells. **c** In vitro editing efficiency for mouse *Hpd* and *Tyr* genes with Un1Cas12f1 in N2a cells. **d** Workflow of embryonic injection of Un1Cas12f1 mRNA and gRNA to generate gene-edited mice. **e** Gene editing efficiency in mice after embryonic injection of Un1Cas12f1 mRNA and gRNA. **f** Schematic map of AAV-CjCas9 and AAV-Un1Cas12f1. **g** Procedure of gene therapy with AAV-CjCas9 and AAV-Un1Cas12f1 in a mouse model of tyrosinaemia. **h** Deep-sequencing results of gene editing outcomes for liver DNA after Cas12f1 treatment. **i** Survival curve for tyrosinaemia mice treated with or without AAV-CjCas9 and AAV-Un1Cas12f1. **j** Mouse weight analysis after treatment with or without AAV-CjCas9 and AAV-Un1Cas12f1. **k** HPD staining results of liver from tyrosinaemia mice treated with or without AAV-CjCas9 and AAV-Un1Cas12f1. All mice were injected with AAV at the age of 8 weeks. Data are represented as means ± SEM. Scale bars, 200 μm.

To further confirm activity of Un1Cas12f1 in mouse cells, we found that at two other genes, *Tyr* and *Hpd*, Un1Cas12f1 could yield 10% and 8% editing efficiency, respectively (Fig. 1c). To examine the use of Cas12f1 in embryos, in vitro transcribed Un1Cas12f1 mRNA and gRNA targeting *Hpd* or *Tyr* gene were introduced into mouse zygotic embryos via microinjection (Fig. 1d; Supplementary Tables S2–S7). Knockout mice generated by injection of *Hpd*-targeting gRNA showed 20% gene editing rate, two times higher than that in N2a cells (Fig. 1e; Supplementary Fig. S1). Similarly, gRNAs targeting *Tyr* gene exhibited up to 20% indel efficiency and robustly disrupted *Tyr* gene as shown by genotyping results (Fig. 1e; Supplementary Fig. S1). Based on results of Cas12f1-mediated editing in mouse N2a cells and embryos, we hypothesize that Cas12f1 can be applied in treating disease via single AAV delivery in adult tissue, given the hypercompact size of Cas12f1.

We explore therapeutic potential of Cas12f1 using a mouse model of tyrosineamia, in which loss-of-function mutations in *Fah* gene result in hereditary tyrosinaemia type I (HTI) and cause lethal liver injury. Inactivation of *4-hydroxyphenylpyruvate dioxygenase*, encoded by *Hpd* gene, can ameliorate the phenotype and convert hepatocytes to a healthy and asymptomatic state^7–9^. Therefore, we select *Hpd* gene as a therapeutic target to test in vivo efficacy of Cas12f1 for HTI treatment. Another small Cas9 nuclease CjCas9 was taken as positive control. As shown in Fig. 1f, Un1Cas12f1 (537 a.a.) is about half the size of the CjCas9 (984 a.a.), the latter of which has previously been identified as the most efficient miniature single effector Cas protein^10^. *Fah*^–/–^ tyrosineamia mice were routinely maintained by NTBC treatment, an HPD inhibitor. To test the efficacy of Cas12f1 in deleting *Hpd*, 7 days before withdrawal of NTBC, AAV-Cas12f1 and AAV-CjCas9 were administered into *Fah*^*–/–*^ tyrosineamia mice via tail vein injection, respectively (Fig. 1g). Mice treated with AAV carrying non-target gRNA succumbed to disease progression 65 days after NTBC withdrawal (Fig. 1i). However, mice treated with AAV-Cas12f1 or AAV-CjCas9 targeting *Hpd* gene survived for more than 75 days after NTBC withdrawal, indicating the comparable treatment efficacy of Cas12f1 and CjCas9 (Fig. 1i). Furthermore, AAV-Cas12f1- or AAV-CjCas9-treated mice exhibited steady increases of body weight during the entire observation period, in contrast to dramatic weight loss in untreated- or vehicle-treated mice (Fig. 1j). Subsequently, we performed genotyping for liver tissue to determine the editing efficiencies of Cas12f1 and CjCas9. Compared with control liver, high percentage of inactivated *Hpd* allele (up to 80%) was found in treated liver, which resulted in a substantial reduction of HPD expression in liver as revealed by immunostaining (Fig. 1h, k; Supplementary Figs. S2, S3), consistent with the phenotype that tyrosineamia symptoms were alleviated after AAV-Cas12f1 or AAV-CjCas9 treatment. Likewise, unlike severe liver fibrosis occurred in untreated mice after NTBC withdrawal, H&E staining showed comparable alleviation of liver damage between Cas12f1- and CjCas9-treated mice (Supplementary Fig. S4). Furthermore, hepatocytes of Cas12f1-treated *Fah*^*–/–*^ mice showed reduced senescence than that of control mice (Supplementary Fig. S5a, b). We also analyzed multiple liver-related metabolic biomarkers, including ALB, AST, ALT, total bilirubin and tyrosine metabolism, to evaluate the overall therapeutic effect of Cas12f1-mediated *Hpd* knockout. Measurements of all serum indicators demonstrated that dysfunctional tyrosine metabolism was significantly rescued after Cas12f1 treatment (Supplementary Fig. S5c–g). To explore the broad utility of Cas12f1 in disease intervention, we next performed Cas12f1-mediated knockout of *Klkb1*, another gene therapy target for treatment of hereditary angioedema. Cas12f1 showed modest knockout efficiency for mouse *Klkb1* gene (Supplementary Fig. S6a, b), indicating the potential use of Cas12f1 as a treatment for *Klkb1* related diseases after optimization of current system in the future. To investigate off-target effect of Cas12f1 in vivo, we predicted and profiled 10 potential off-target sites with OFFinder and amplicon sequencing for Cas12f1 and CjCas9. We observed no detectable indel mutation at predicted off-target loci in either Cas12f1 or CjCas9 treatment group (Supplementary Figs. S3, S4, S7–S9). Moreover, we conducted genome-wide off-target analysis using PEM-seq to evaluate off-target effect induced by Cas12f1. PEM-seq did not detect off-target editing events in Cas12f1-mediated gene knockout (Supplementary Fig. S10). These results suggested that Cas12f1 showed comparable efficacy and off-target profile with CjCas9 in rescuing liver phenotype in this mouse model of tyrosinaemia. Overall, our finding showed proof-of-concept evidence that Un1Cas12f1 can be applied in generating genetically modified animal models and in treating hereditary diseases given its size advantage over other Cas proteins.

Although increasing number of different Cas9 and Cas12 orthologs were identified^11^, most of orthologs consist of ~1000 a.a., making it cumbersome for their delivery via AAV. Lately, Cas12f1 with only ~500 a.a. was demonstrated to have nuclease activity in eukaryotic cells, showing its great potential as an AAV-based gene therapy system. Our present study demonstrated the use of Cas12f1 to correct a mouse model of tyrosineamia. To broaden the applications of Cas12f1, it would be necessary to further improve gene editing activity and reduce complexity of PAM sequences that enables more genomic loci editable. Though no obvious off-target and toxicity were observed in our study, long-term evaluation of both off-target and other safety issues are warranted before Cas12f1 can be used in human settings. Given its hypercompact size, these findings highlight the potential of in vivo editing by Un1Cas12f1 as a possible treatment for genetic diseases.

## Supplementary information

Supplementary Information

## Data Availability

The deep-sequencing data in this study have been deposited in the NCBI Sequence Read Archive (SRA) BioProject database under accession number PRJNA963402. Plasmids are available from the corresponding authors upon request.
