# Supplementary material for: In vivo treatment of tyrosinaemia with hypercompact Cas12f1
Source: Cell Discov. 2023 Jul 18;9:73. doi: 10.1038/s41421-023-00554-y (PMC10352374; doi:10.1038/s41421-023-00554-y)
Supplement: Supplementary file 1 — Supplementary Information [file 41421_2023_554_MOESM1_ESM.pdf]

Supplementary materials for

In vivo treatment of tyrosinaemia with hypercompact Cas12f1

Ruochen Guo[1,2†], Zhifang Li[1,3†], Guoling Li[2†], Hainan Zhang[4†], Chang Zhang[2†], Xiaona Huo[1,3], Xiaoyin Zhang[1,3], Xiali Yang[2], Rongrong Yang[1,3], Yuanhua Liu[2], Xiaozhi Sun[1,5], Xinyu Liu[2], Hui Yang[2,3,4*], Yingsi Zhou[4*], Chunlong Xu[1,3,5*]

†These authors contributed equally to this work.
***Corresponding author Email:** huiyang@huidagene.com; yingsizhou@huidagene.com;xucl@lglab.ac.cn

This PDF file includes:

Materials and Methods
Figure S1 Genotyping results for offspring from Cas12f1-edited mouse embryos.
Figure S2 On-target editing analysis of Hpd gene for mouse liver DNA after Un1Cas12f1 treatment.
Figure S3 On-target editing analysis of Hpd gene for mouse liver DNA after CjCas9 treatment.
Figure S4 H&E analysis of liver for Cas12f1 and CjCas9-treated mice.
Figure S5 Phenotypic evaluation of Cas12f1-treated Fah-/- mice.
Figure S6 Klkb1 knockout via Cas12f1.
Figure S7 Off-target analysis of predicted off-target loci after Un1Cas12f1 treatment.
Figure S8 Off-target analysis of predicted off-target loci after CjCas9 treatment.
Figure S9 Off-target analysis of predicted off-target loci after Klkb1 editing by Un1Cas12f1.
Figure S10 Genome-wide off-target analysis for Un1Cas12f1 with PEM-seq.
Table S1 Summary table of gRNA tested with or without editing activity for three different Cas12f1 orthologs
Table S2-7 gRNA and primer sequence

Materials and Methods

Study approval

The objectives of the present study were to show proof-of-concept evidence for in vivo therapeutic efficacy of Cas12f1 delivered by AAV. All animal experiments were approved by the Animal Care and Use Committee of Shanghai Center for Brain Science and Brain-Inspired Technology, Shanghai, China.

Plasmid constructions

The pCAG-Un1Cas12f1-hU6-BpiI plasmid encoded a human codon-optimized Un1Cas12f1 driven by CAG promoter, and hU6-driven crRNAs with *Bpi*I cloning site. The sgRNAs were designed suitable for Un1Cas12f1 and CjCas9, then synthesized as DNA oligonucleotides and cloned into pCAG-Un1Cas12f1 to form the CRISPR targeting plasmids (listed in Supplementary Tables S2-7).

Cell culture, transfection and flow cytometry analysis

HEK293T were maintained in Dulbecco's modified eagle medium (DMEM) (Gibco, 11965092) supplemented with 10% fetal bovine serum at 37 °C and 5% CO_2 in a humidified incubator. For sgRNA screening, CRISPR targeting plasmids and reporter were co-transfected using polyethylenimine (PEI) transfection reagent. After transfected cells were cultured with 48 hours, we carefully resuspended the cell pellet, and then analyzed or sorted by BD FACSAria II. Flow cytometry results were analyzed with FlowJo X (v.10.0.7).

In vitro transcription of Un1Cas12f1 and sgRNA

Un1Cas12f1 mRNA was transcribed using the mMESSAGE mMACHINE T7 Ultra Kit(Invitrogen, AM1345). T7 promoter was added to sgRNA template by PCR amplification of pCX1991 using primer F and R. The PCR products purified with Omega gel extraction Kit(Omega, D2500-02), templates were

transcribed using the MEGAshortscript Kit(Invitrogen, AM1354). The un1Cas12f1 mRNA and sgRNAs were purified by MEGAclear Kit(Invitrogen, AM1908), eluted with RNase-free water and stored at -80°C.

Zygote injection and embryo transplantation

Eight-week-old B6D2F1 female mice were super ovulated and mate with B6D2F1 male mice, and fertilized embryos were collected from oviduct. The mixture of un1Cas12f1 mRNA(100ng/μl) and sgRNA(100ng/μl) was injected into the cytoplasm of fertilized eggs using a FemtoJet microinjector(Eppendrof). The injected embryos were cultured in KOSM medium with amino acids at 37°C under 5% CO_2 in a humidified incubator overnight and then transferred into oviducts of pseudo-pregnant ICR foster mothers at 0.5-d.p.c.

Mice and hydrodynamic injection

All animal study protocols were approved by the National Institutes of Health (NIH) Guide for the Care and Use of Laboratory Animals. Fahmut/mut mice were kept on 10mg/L NTBC (Sigma-Aldrich, Cat. No. PHR1731) in drinking water when indicated. For hydrodynamic liver injection, AAV8 (4×10^{11} vg/mouse) in 200 μl saline were injected via the tail vein into 8-10 weeks old male and female mice. Mice were kept off NTBC water at 7 days post injection, and their body weights were recorded every 3-5 days. Mice were harvested at 75 days after NTBC water withdrawal for histology and DNA analysis. Control mice off NTBC water were harvested when reaching >20% weight loss.

AAV virus production

The adeno-associated virus 8 (AAV8) serotype was used in this study. The Cas12f1 or Cjcas9 plasmids with target or nontarget gRNA was sequenced before packaging into AAV8 vehicle, and the AAV vectors were packaged by

PackGene Biotech. The virus titer was 2.57 × 10^{13} (Ad-Cas12f1-Hpd), 2.32 × 10^{13} (Ad-Cas12f1-Null), 2.62 × 10^{13} (Ad-CjCas9-Hpd) and 1.93× 1013 (Ad-CjCas9-Null) genome copies/ml as determined by qPCR specific for the inverted terminal repeat.

Histological analysis and Serum biochemistry

Liver tissues were harvested, and sections were fixed in 4% PFA overnight. The following antibodies were used: anti-HPD antibody (SantaCruz, sc-390279; dilution 1:100), anti-P21 antibody (Abcam, ab109199; dilution 1:200). Immunohistochemistry, immunofluorescence and hematoxylin and eosin (H&E) staining were performed by the standard procedures. Blood was collected using retro-orbital puncture before mice was sacrificed. ALT, AST, ALB, tyrosine and bilirubin levels in serum were determined using diagnostic ELISA Kits (Abcam, HWRK chem).

Targeted deep sequencing

To analyze un1Cas12f1 targeting efficiency, the DNA of successfully transfected cells or AAV8 treatment tissues were extracted with TIANamp Genomic DNA Kit(TIANGEN,) according to the manufacturer protocol. DNA was amplified with Phanta max super-fidelity DNA polymerase (Vazyme, P505-d1) for Sanger or deep sequencing methods. And deep sequencing libraries were used to add Illumina flow cell binding sequences and specific barcodes on the 5′ and 3' end of the primer sequence. The products were pooled and sequenced with 150 paired-end reads on an Illumina Hiseq instrument. FASTQ format data were analyzed using the Cutadapt (v.2.8)41 according to assigned barcode sequences.

PEM-seq analysis

Genome-wide off-target analysis was performed following PEM-seq protocol previously described[12]. The 20 μg genomic DNA from Un1Cas12f1 edited or

control samples were fragmented with Covaris sonicator to generate 300-700 bp DNA. DNA fragments was tagged with biotin at 5'-end by one-round PCR extension using a biotinylated primer, primer leftover removed by AMPure XP beads and purified by streptavidin beads. The single-stranded DNA on streptavidin beads is ligated with a bridge adapter containing 14-bp random molecular barcode, and PCR product was generated via nested PCR to enrich DNA fragment containing the bait DSB events and tagged with illumine adapter sequences. The prepared sequencing library was sequenced by Hi-seq 2500 with 150 bp pair-end reads.

Statistical analysis

The number of independent biological replicates were shown in the figure legend. The data are presented as means ± SEM. Differences were assessed using unpaired two-tailed Student's t tests. Differences in means were considered statistically significant at $P < 0.05$.

Figure S1

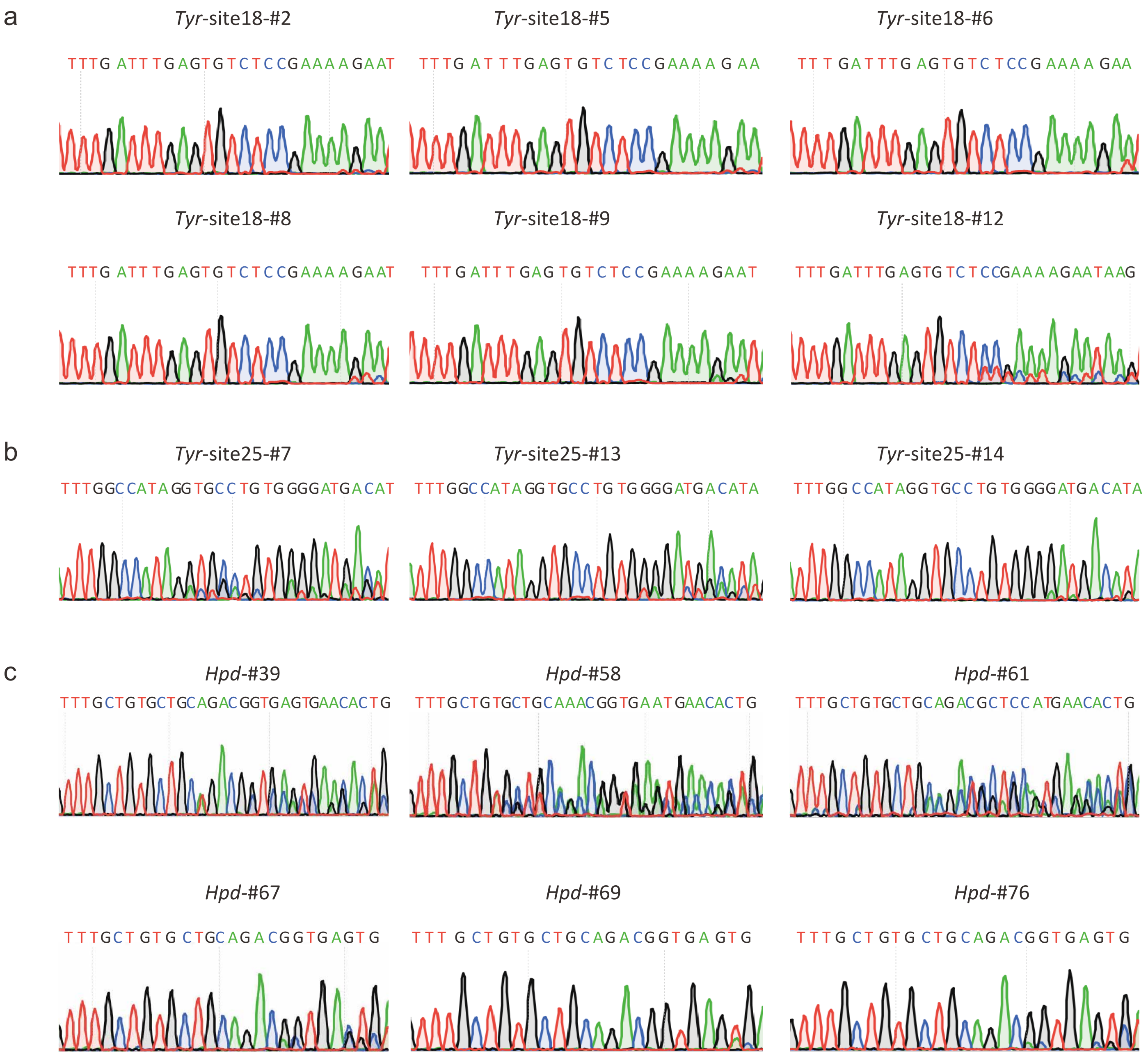

a
Tyr-site18-#2
TTTGATTTGAGTGTCTCCGAAAAGAAT
Tyr-site18-#5
TTTGATTTGAGTGTCTCCGAAAAGAA
Tyr-site18-#6
TTTGATTTGAGTGTCTCCGAAAAGAA
Tyr-site18-#8
TTTGATTTGAGTGTCTCCGAAAAGAAT
Tyr-site18-#9
TTTGATTTGAGTGTCTCCGAAAAGAAT
Tyr-site18-#12
TTTGATTTGAGTGTCTCCGAAAAGAATAAG
b
Tyr-site25-#7
TTTGGCCATAGGTGCCTGTGGGGATGACAT
Tyr-site25-#13
TTTGGCCATAGGTGCCTGTGGGGATGACATA
Tyr-site25-#14
TTTGGCCATAGGTGCCTGTGGGGATGACATA
c
Hpd-#39
TTTGCTGTGCTGCAGACGGTGAGTGAACACTG
Hpd-#58
TTTGCTGTGCTGCAAACGGTGAATGAACACTG
Hpd-#61
TTTGCTGTGCTGCAGACGCTCCATGAACACTG
Hpd-#67
TTTGCTGTGCTGCAGACGGTGAGTG
Hpd-#69
TTTGCTGTGCTGCAGACGGTGAGTG
Hpd-#76
TTTGCTGTGCTGCAGACGGTGAGTG

Supplementary figures & legend

Fig. S1 Genotyping results for offspring from Cas12f1-edited mouse embryos. a Sanger sequencing results for site 18 in *Tyr* gene of offspring derived from Cas12f1-injected embryos. **b** Sanger sequencing results for site-25 in *Tyr* gene of offspring derived from Cas12f1-injected embryos. **c** Sanger sequencing results for site-39 in *Hpd* gene of offspring derived from Cas12f1-injected embryos.

Figure S2

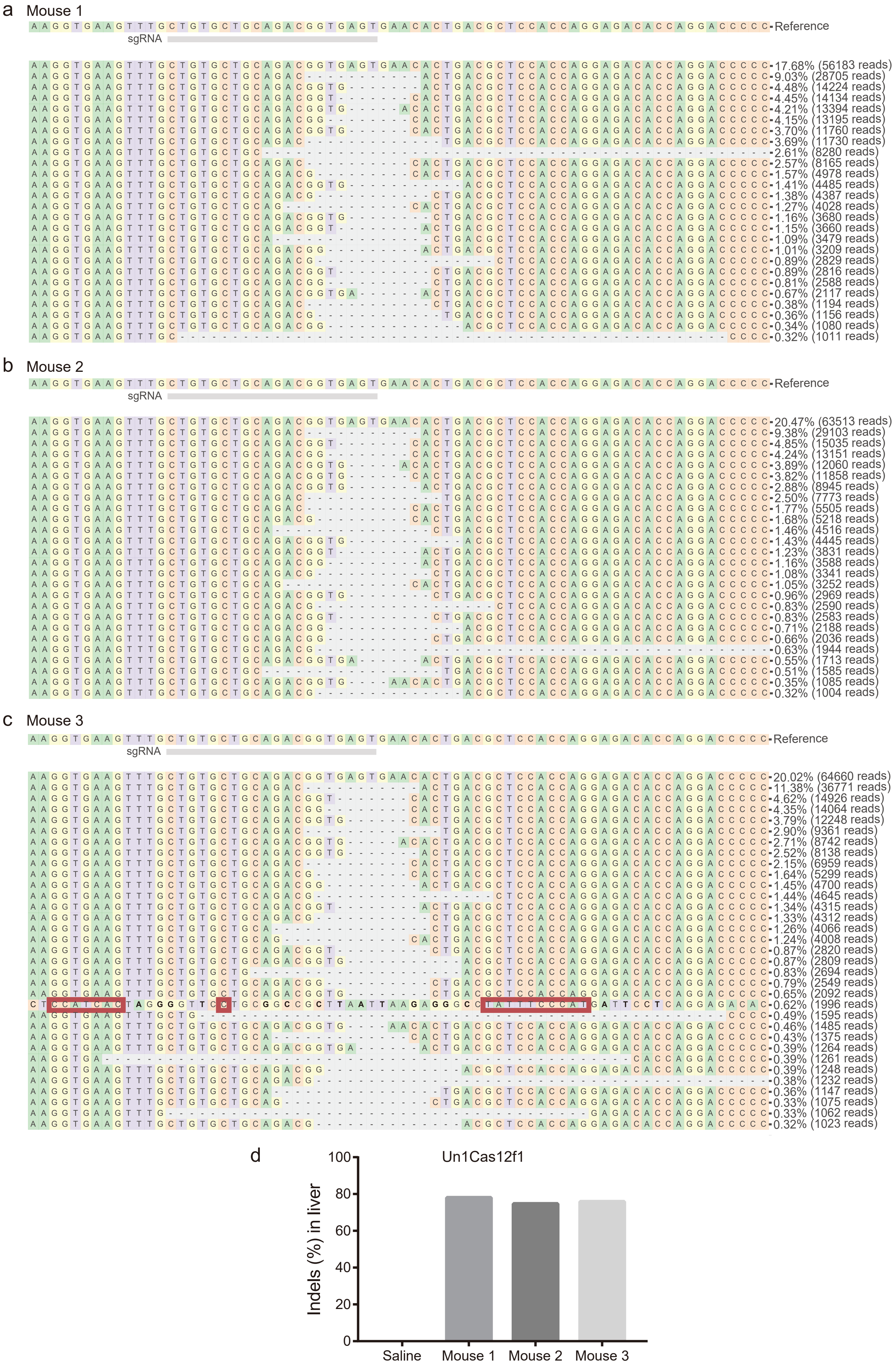
a
Mouse 1
AAGGTGAAGTTTGCTGTGCTGCAGACGGTGAGTGAACACTGACGCTCCACCAGGAGACACCAGGACCCCC
Reference
sgRNA
17.68% (56183 reads)
9.03% (28705 reads)
4.48% (14224 reads)
4.45% (14134 reads)
4.21% (13394 reads)
4.15% (13195 reads)
3.70% (11760 reads)
3.69% (11730 reads)
2.61% (8280 reads)
2.57% (8165 reads)
1.57% (4978 reads)
1.41% (4485 reads)
1.38% (4387 reads)
1.27% (4028 reads)
1.16% (3680 reads)
1.15% (3660 reads)
1.09% (3479 reads)
1.01% (3209 reads)
0.89% (2829 reads)
0.89% (2816 reads)
0.81% (2588 reads)
0.67% (2117 reads)
0.38% (1194 reads)
0.36% (1156 reads)
0.34% (1080 reads)
0.32% (1011 reads)
b
Mouse 2
AAGGTGAAGTTTGCTGTGCTGCAGACGGTGAGTGAACACTGACGCTCCACCAGGAGACACCAGGACCCCC
Reference
sgRNA
20.47% (63513 reads)
9.38% (29103 reads)
4.85% (15035 reads)
4.24% (13151 reads)
3.89% (12060 reads)
3.82% (11858 reads)
2.88% (8945 reads)
2.50% (7773 reads)
1.77% (5505 reads)
1.68% (5218 reads)
1.46% (4516 reads)
1.43% (4445 reads)
1.23% (3831 reads)
1.16% (3588 reads)
1.08% (3341 reads)
1.05% (3252 reads)
0.96% (2969 reads)
0.83% (2590 reads)
0.83% (2583 reads)
0.71% (2188 reads)
0.66% (2036 reads)
0.63% (1944 reads)
0.55% (1713 reads)
0.51% (1585 reads)
0.35% (1085 reads)
0.32% (1004 reads)
c
Mouse 3
AAGGTGAAGTTTGCTGTGCTGCAGACGGTGAGTGAACACTGACGCTCCACCAGGAGACACCAGGACCCCC
Reference
sgRNA
20.02% (64660 reads)
11.38% (36771 reads)
4.62% (14926 reads)
4.35% (14064 reads)
3.79% (12248 reads)
2.90% (9361 reads)
2.71% (8742 reads)
2.52% (8138 reads)
2.15% (6959 reads)
1.64% (5299 reads)
1.45% (4700 reads)
1.44% (4645 reads)
1.34% (4315 reads)
1.33% (4312 reads)
1.26% (4066 reads)
1.24% (4008 reads)
0.87% (2820 reads)
0.87% (2809 reads)
0.83% (2694 reads)
0.79% (2549 reads)
0.65% (2092 reads)
0.62% (1996 reads)
0.49% (1595 reads)
0.46% (1485 reads)
0.43% (1375 reads)
0.39% (1264 reads)
0.39% (1261 reads)
0.39% (1248 reads)
0.38% (1232 reads)
0.36% (1147 reads)
0.33% (1075 reads)
0.33% (1062 reads)
0.32% (1023 reads)
d
Un1Cas12f1
Indels (%) in liver
100
80
60
40
20
0
Saline
Mouse 1
Mouse 2
Mouse 3

Fig. S2 On-target editing analysis of *Hpd* gene for mouse liver DNA after Un1Cas12f1 treatment. a-c Deep-seq results for Hpd gene editing in three Un1Cas12f1-treated mice. **d** Percentage of Indel mutations induced by AAV-Un1Cas12f1 in three mice.

Figure S3

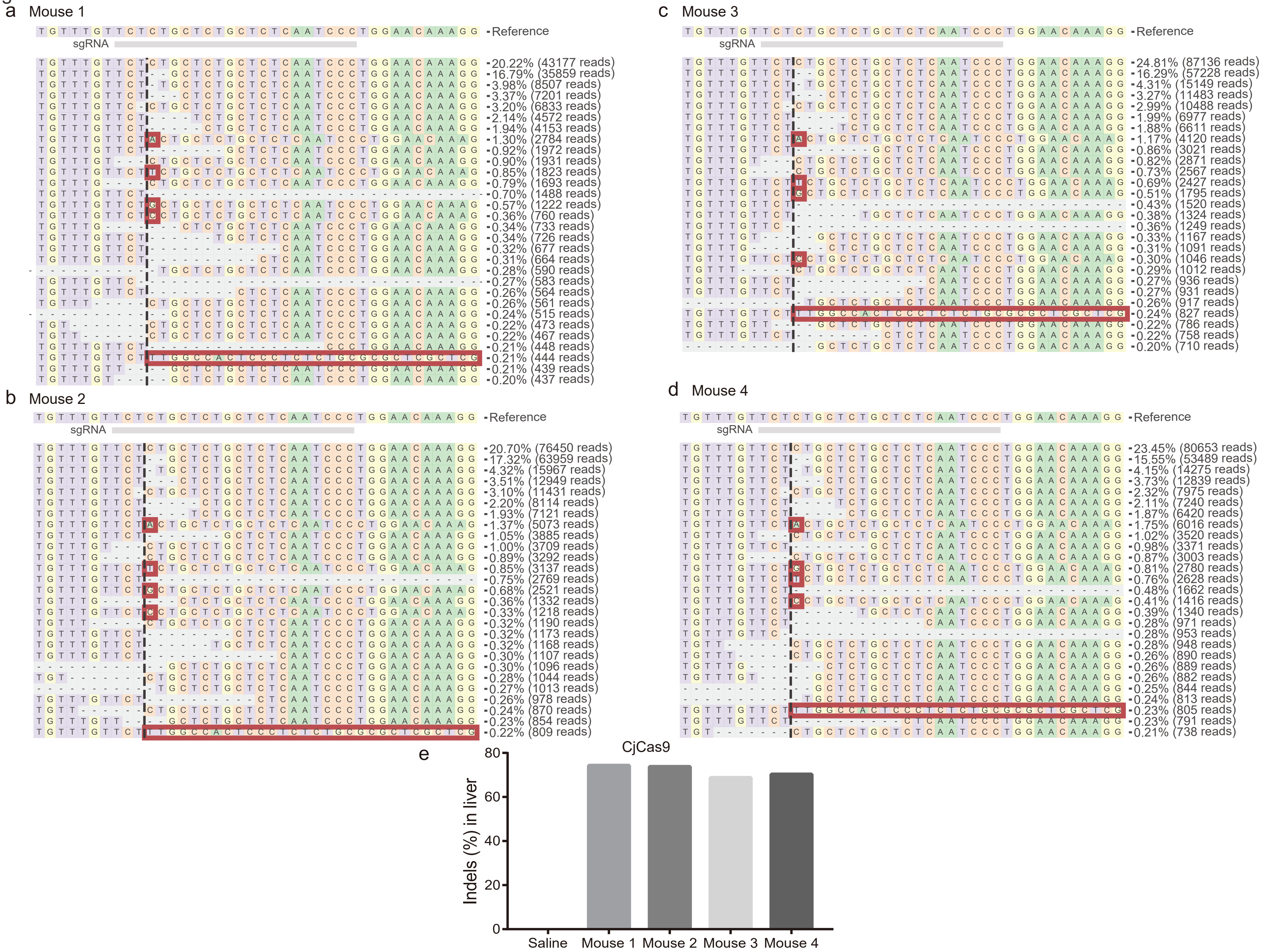
a Mouse 1
b Mouse 2
c Mouse 3
d Mouse 4
Reference
sgRNA
e
CjCas9
Indels (%) in liver
Saline
Mouse 1
Mouse 2
Mouse 3
Mouse 4

Fig. S3 On-target editing analysis of *Hpd* gene for mouse liver DNA after CjCas9 treatment. a-d Deep-seq results for Hpd gene editing in four CjCas9-treated mice. **e** Percentage of Indel mutations induced by AAV- CjCas9 in four mice.

Figure S4

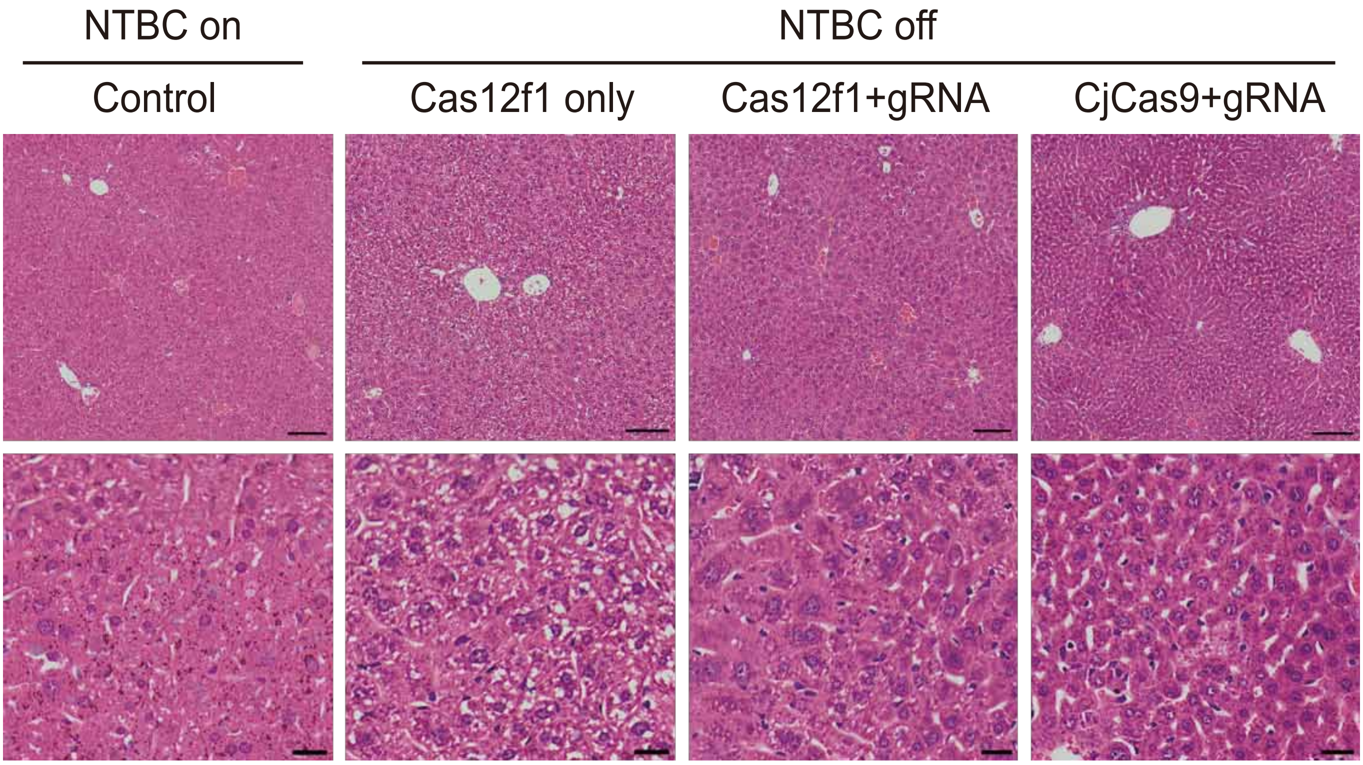

NTBC on
NTBC off
Control
Cas12f1 only
Cas12f1+gRNA
CjCas9+gRNA

Fig. S4 H&E analysis of liver for Cas12f1 and CjCas9-treated mice. Control represents liver from mice maintained with NTBC and without treatment. Cas12f1 only group indicate liver from mice treated with Cas12f1 and non-target RNA. Cas12f1 + gRNA and CjCas9 + gRNA represent liver from mice treated with Cas protein and gRNA against *Hpd* gene.

Figure S5

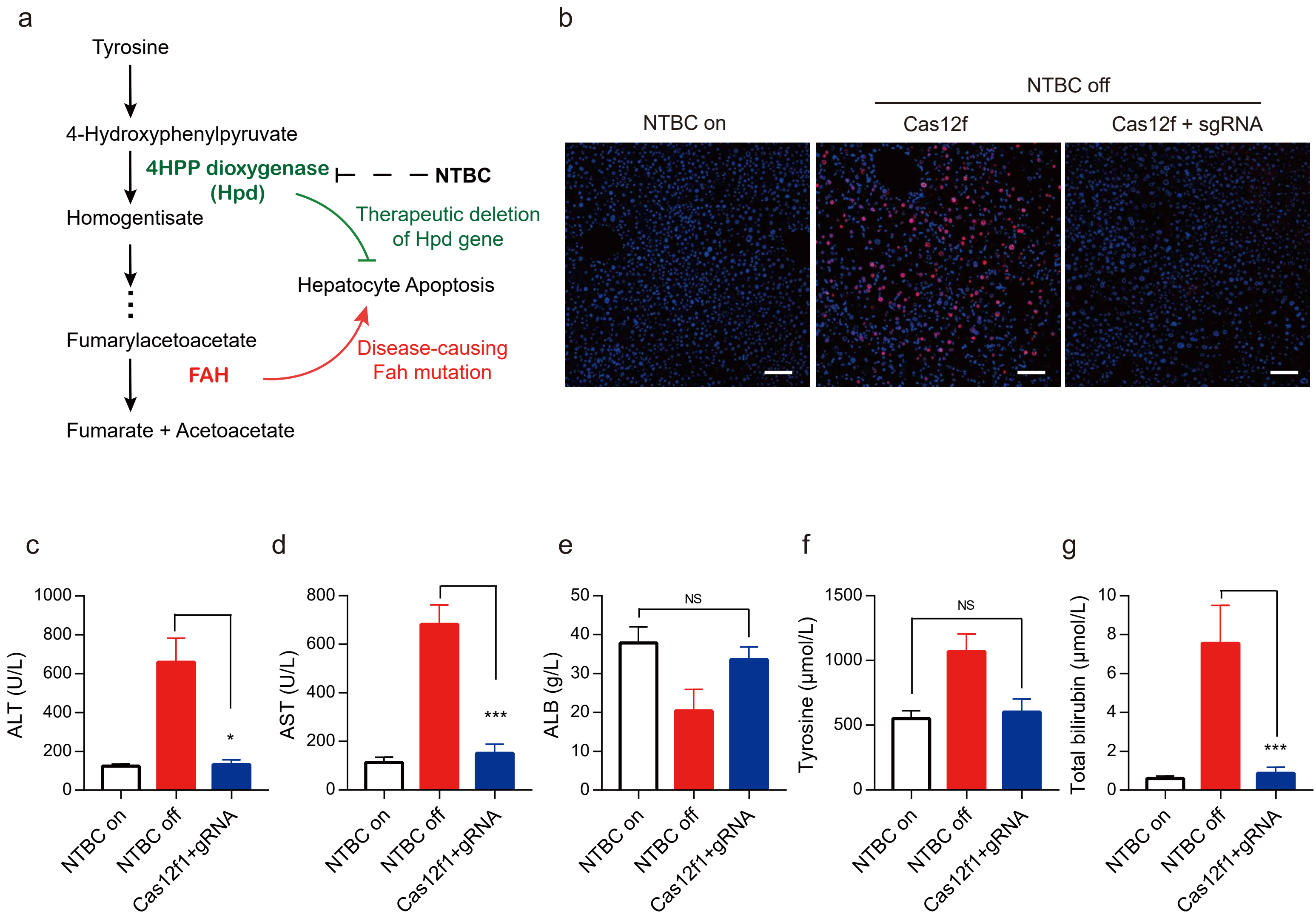
a
Tyrosine
4-Hydroxyphenylpyruvate
4HPP dioxygenase (Hpd)
NTBC
Homogentisate
Therapeutic deletion of Hpd gene
Hepatocyte Apoptosis
Fumarylacetoacetate
FAH
Disease-causing Fah mutation
Fumarate + Acetoacetate
b
NTBC off
NTBC on
Cas12f
Cas12f + sgRNA
c
ALT (U/L)
d
AST (U/L)
e
ALB (g/L)
NS
f
Tyrosine (μmol/L)
NS
g
Total bilirubin (μmol/L)
*
***
***
NTBC on
NTBC off
Cas12f1+gRNA

Fig. S5 Phenotypic evaluation of Cas12f1-treated Fah-/- mice. a Tyrosine metabolism pathway. **b** P21 staining results for mouse liver with or without Cas12f1 treatment (30 days post NTBC off). Red and blue indicate P21 and DAPI signal respectively. **c-f** Biochemical analysis of serum indicators for liver metabolic function in Cas12f1-treated or untreated mice (n=3). Liver damage markers aspartate aminotransferase (AST), alanine aminotransferase (ALT), Albumin (ALB), bilirubin and tyrosine were measured in peripheral blood from Fah-/- mice injected with Cas12f without or with gRNA (NTBC off, day 30). Fah-/- mice on NTBC water (NTBC on) served as a control. Data are represented as means ± SEM. Unpaired two-tailed Student's t tests. * $P < 0.05$, *** $P < 0.001$, NS non-significant. Scale bars, 100 μm.

Figure S6

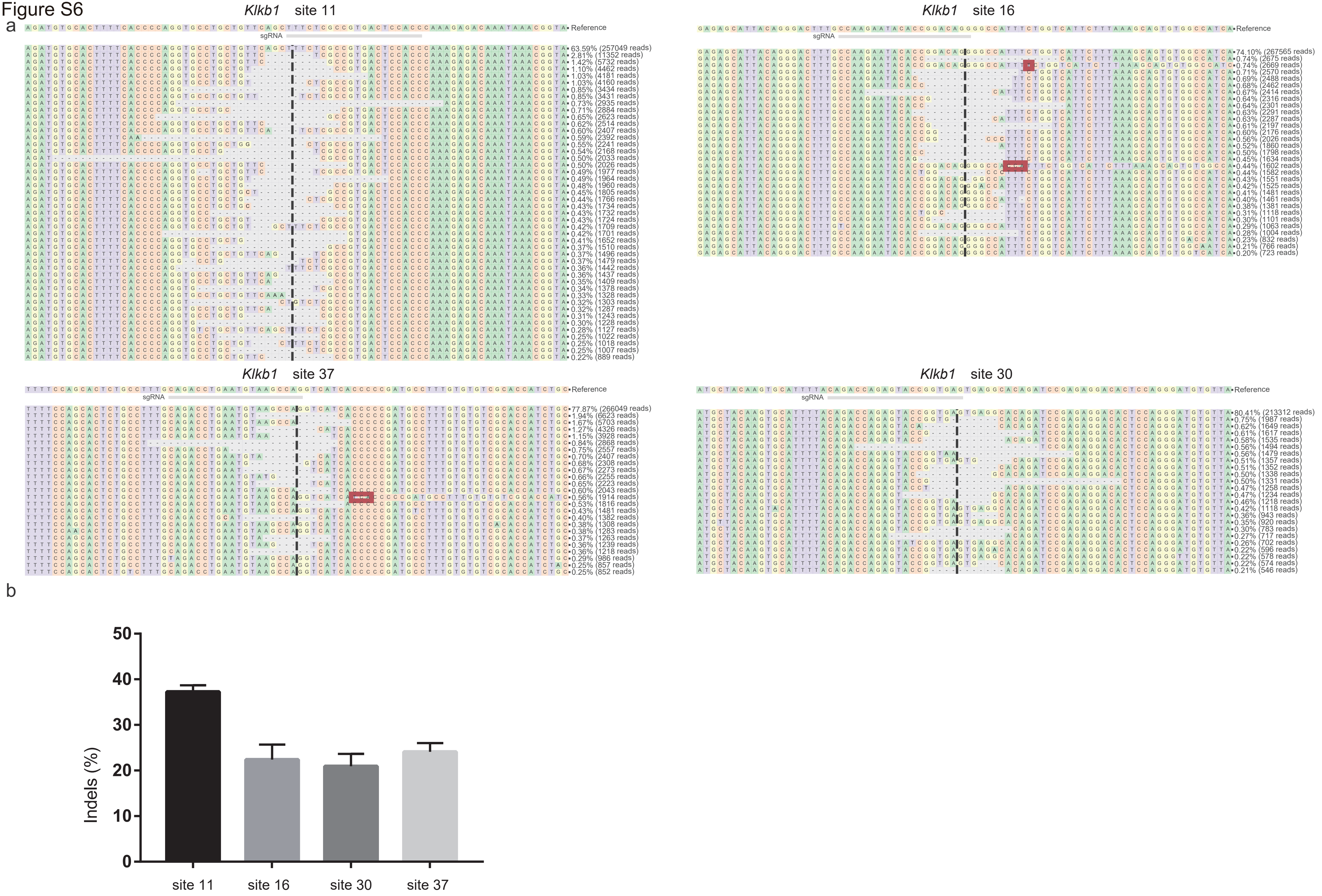
a
Klkb1 site 11
Klkb1 site 16
Klkb1 site 37
Klkb1 site 30
Reference
sgRNA
b
Indels (%)
50
40
30
20
10
0
site 11
site 16
site 30
site 37

Fig. S6 Klkb1 knockout via Cas12f1. a Indel mutations for Klkb1 knockout by Cas12f1. **b** Knockout efficiency for Klkb1.

Figure S7

Off-target analysis for Un1Cas12f1-edited liver

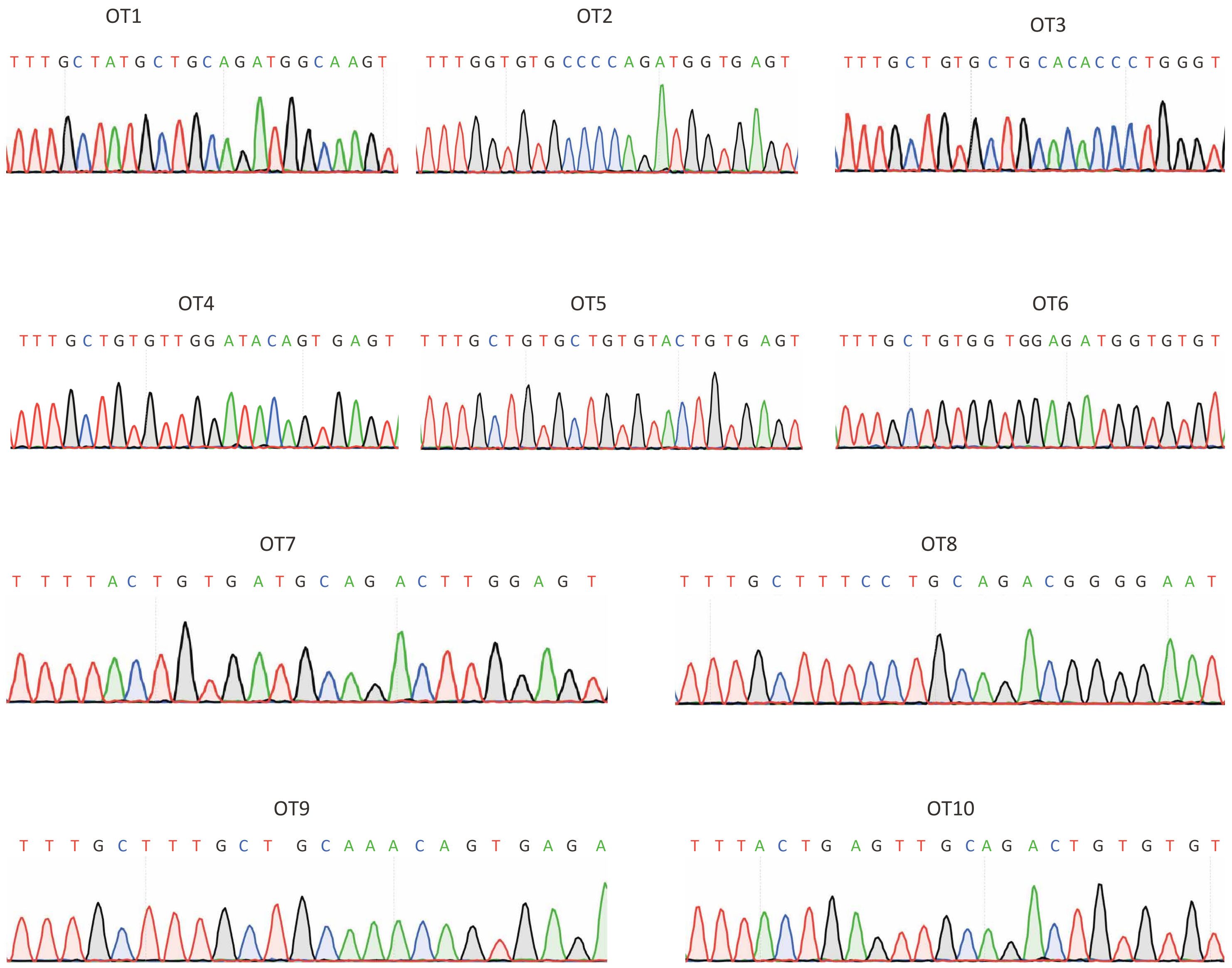

Fig. S7 Off-target analysis of predicted off-target loci after Un1Cas12f1 treatment. Sanger sequencing results were shown for top 10 off-target loci (OT1 to OT10) predicted by OFFinder. OT: off-target.

Figure S8

Off-target analysis for CjCas9-edited liver

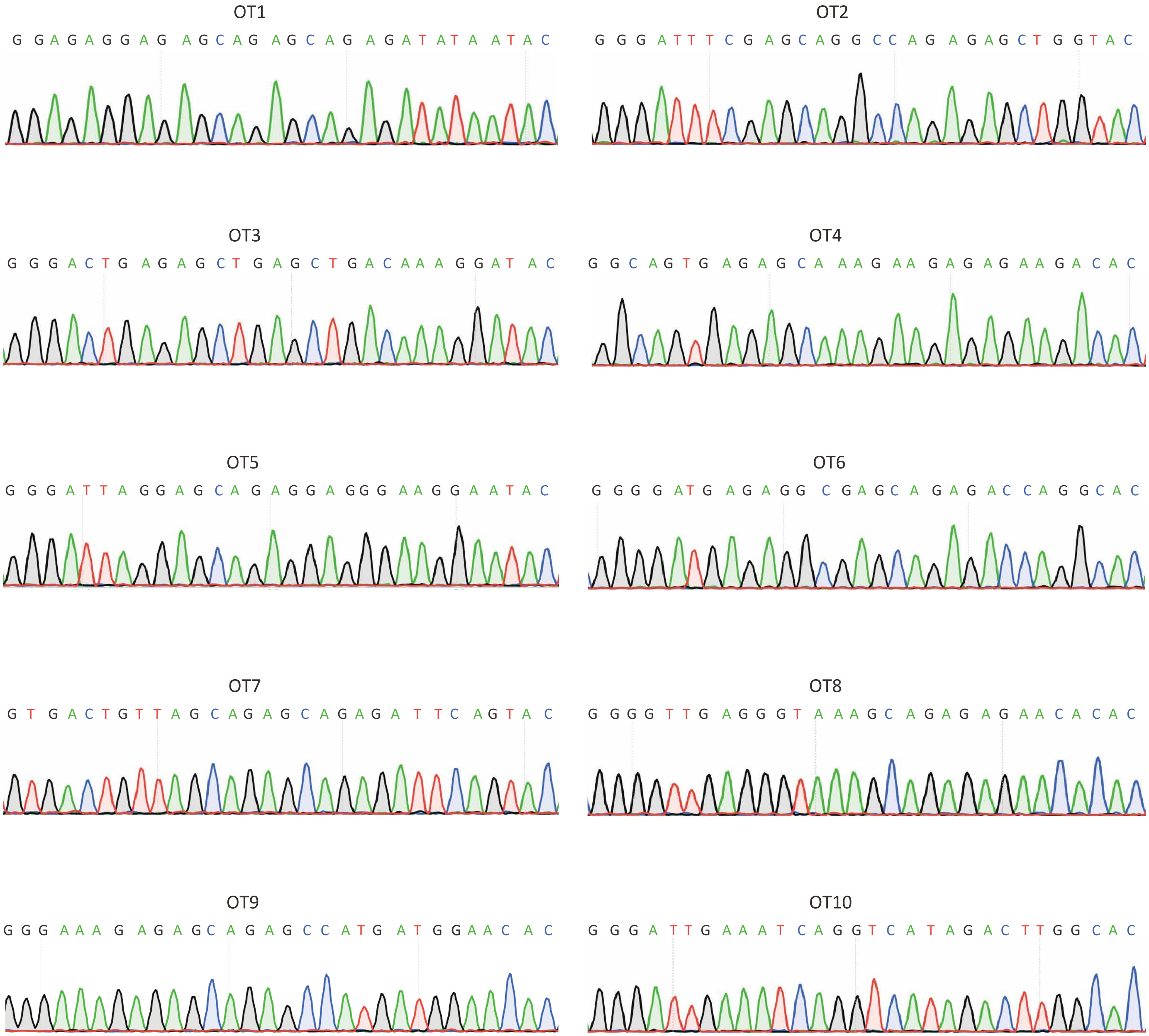

Fig. S8 Off-target analysis of predicted off-target loci after CjCas9 treatment. Sanger sequencing results were shown for top 10 off-target loci (OT1 to OT10) predicted by OFFinder. OT: off-target.

Figure S9

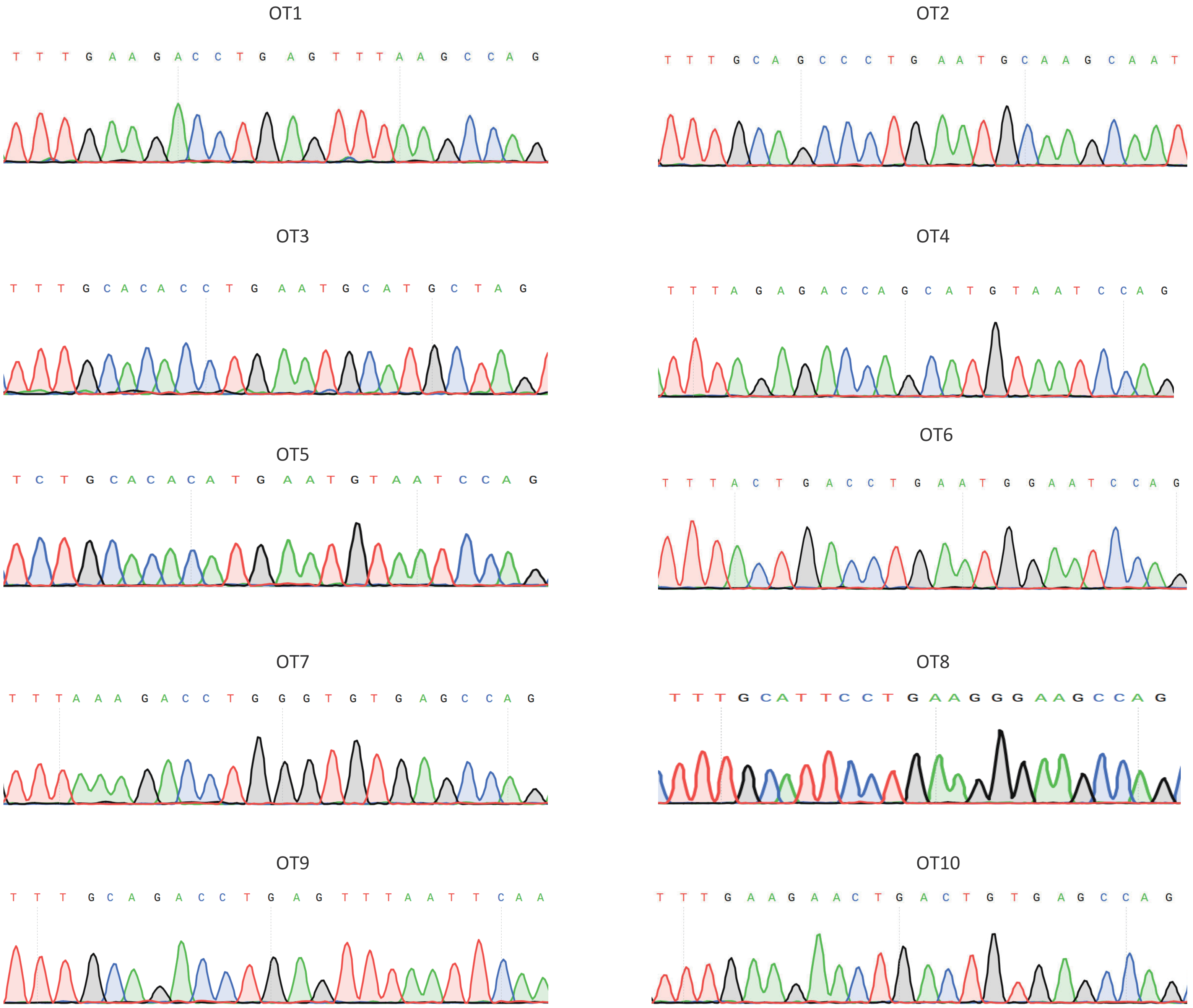
OT1
T T T G A A G A C C T G A G T T T A A G C C A G
OT2
T T T G C A G C C C T G A A T G C A A G C A A T
OT3
T T T G C A C A C C T G A A T G C A T G C T A G
OT4
T T T A G A G A C C A G C A T G T A A T C C A G
OT5
T C T G C A C A C A T G A A T G T A A T C C A G
OT6
T T T A C T G A C C T G A A T G G A A T C C A G
OT7
T T T A A A G A C C T G G G T G T G A G C C A G
OT8
T T T G C A T T C C T G A A G G G A A G C C A G
OT9
T T T G C A G A C C T G A G T T T A A T T C A A
OT10
T T T G A A G A A C T G A C T G T G A G C C A G

Fig. S9 Off-target analysis of predicted off-target loci after Klkb1 editing by Un1Cas12f1. Sanger sequencing results were shown for top 10 off-target loci (OT1 to OT10) predicted by OFFinder. OT: off-target.

Figure S10

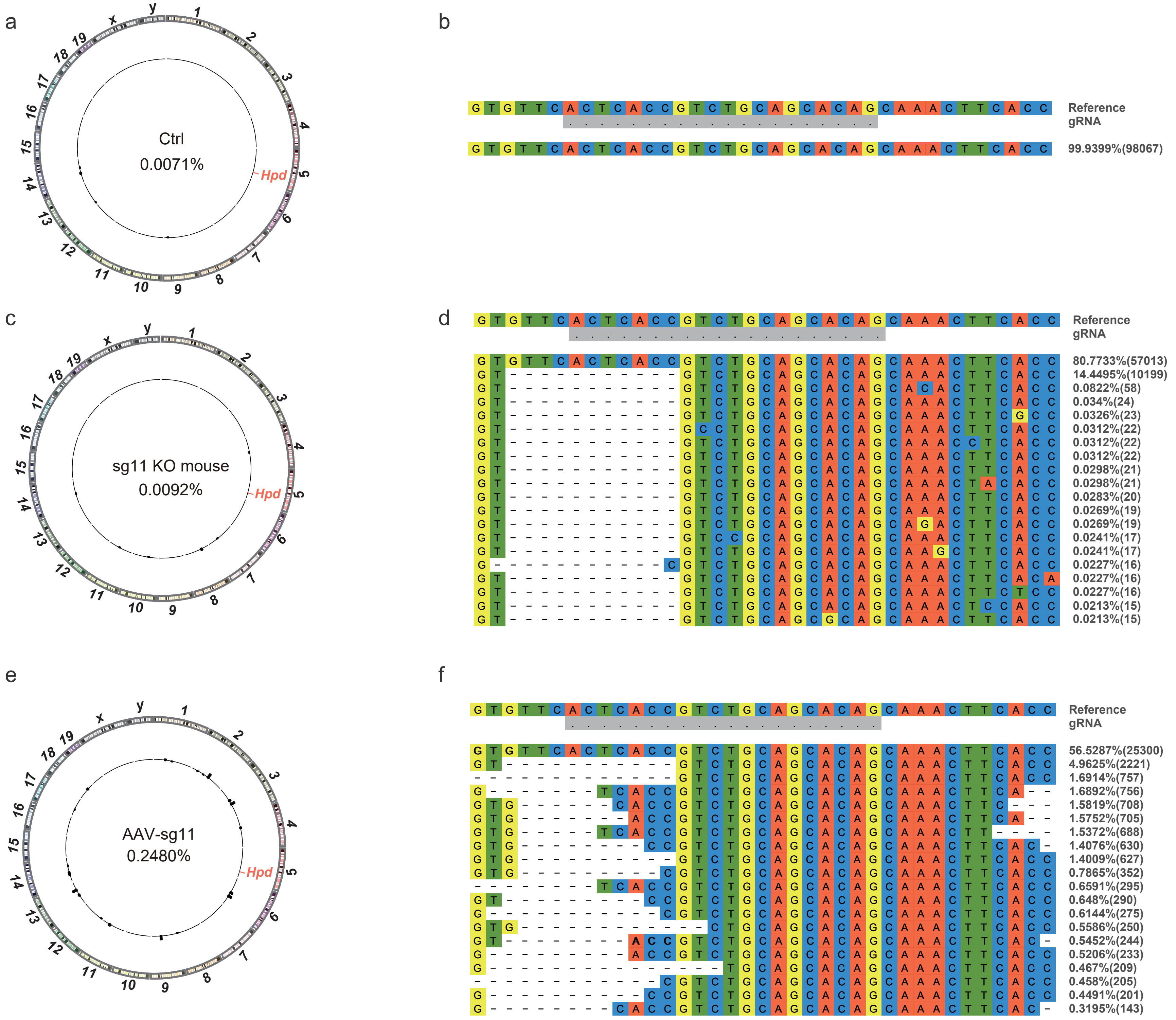

a
Ctrl
0.0071%
Hpd
b
GTGTTCACTCACCGTCTGCAGCACAGCAAACTTCACC
Reference
gRNA
99.9399%(98067)
c
sg11 KO mouse
0.0092%
Hpd
d
Reference
gRNA
80.7733%(57013)
14.4495%(10199)
0.0822%(58)
0.034%(24)
0.0326%(23)
0.0312%(22)
0.0312%(22)
0.0312%(22)
0.0298%(21)
0.0298%(21)
0.0283%(20)
0.0269%(19)
0.0269%(19)
0.0241%(17)
0.0241%(17)
0.0227%(16)
0.0227%(16)
0.0227%(16)
0.0213%(15)
0.0213%(15)
e
AAV-sg11
0.2480%
Hpd
f
Reference
gRNA
56.5287%(25300)
4.9625%(2221)
1.6914%(757)
1.6892%(756)
1.5819%(708)
1.5752%(705)
1.5372%(688)
1.4076%(630)
1.4009%(627)
0.7865%(352)
0.6591%(295)
0.648%(290)
0.6144%(275)
0.5586%(250)
0.5452%(244)
0.5206%(233)
0.467%(209)
0.458%(205)
0.4491%(201)
0.3195%(143)

Fig. S10 Genome-wide off-target analysis for Un1Cas12f1 with PEM-seq. **a,c,e** No genome-wide translocation events detected by PEM-seq for unedited (a) versus edited mouse tissue via embryonic injection (c) or AAV transduction (e) of Un1Cas12f1 targeting Hpd. **b,d,f** Indel mutations detected in unedited (b) versus edited mouse tissue (d,f) by PEM-seq.

Supplementary Tables

Table S1. Summary table of gRNA tested with or without editing activity for three different Cas12f1 orthologs

Gene editing tool	Number of tested site	Number of effective editing site
AsCas12f1	30	0
SpCas12f1	29	0
Un1Cas12f1	32	5

Table S2. AsCas12f1 target site sequences in this study.

sgRNA	Target site sequences (5'-3')	oligos
Trp53-site1	CTGCCCCAGGATGTTGAGGA	GAAC CTGCCCCAGGATGTTGAGGA
		AAAA TCCTCAACATCCTGGGGCAG
Trp53-site2	AGGAGTTTTTTGAAGGCCCA	GAAC AGGAGTTTTTTGAAGGCCCA
		AAAA TGGGCCTTCAAAAAACTCCT
Trp53-site3	AAGGCCCAAGTGAAGCCCTC	GAAC AAGGCCCAAGTGAAGCCCTC
		AAAA GAGGGCTTCACTTGGGCCTT
Trp53-site4	GGCCTTCAAAAAACTCCTCA	GAAC GGCCTTCAAAAAACTCCTCA
		AAAA TGAGGAGTTTTTTGAAGGCC
Trp53-site5	TCCCTTCTCAAAAAACTTAC	GAAC TCCCTTCTCAAAAAACTTAC
		AAAA GTAAGTTTTTTGAGAAGGGA
Trp53-site6	AGAAGGGACAAAAGATGACA	GAAC AGAAGGGACAAAAGATGACA
		AAAA TGTCATCTTTTGTCCCTTCT
Trp53-site7	GCTGTCCCAGACTGCAGGAA	GAAC GCTGTCCCAGACTGCAGGAA
		AAAA TTCCTGCAGTCTGGGACAGC
Trp53-site8	TGTGCACGGTGAGTGGGCCC	GAAC TGTGCACGGTGAGTGGGCCC
		AAAA GGGCCCACTCACCGTGCACA
Trp53-site9	AGGGGAGGAGAGTACTGGAA	GAAC AGGGGAGGAGAGTACTGGAA
		AAAA TTCCAGTACTCTCCTCCCCT

Trp53-site10	TGGGTCAGCGCCACACCTCC	GAAC TGGGTCAGCGCCACACCTCC
		AAAA GGAGGTGTGGCGCTGACCCA
Trp53-site11	TAGATGGCCATGGCGCGGAC	GAAC TAGATGGCCATGGCGCGGAC
		AAAA GTCCGCGCCATGGCCATCTA
Trp53-site12	TCCGGGTGGAAGGAAATTTG	GAAC TCCGGGTGGAAGGAAATTTG
		AAAA CAAATTTCCTTCCACCCGGA
Trp53-site13	TATCCCGAGTATCTGGAAGA	GAAC TATCCCGAGTATCTGGAAGA
		AAAA TCTTCCAGATACTCGGGATA
Trp53-site14	TGAGCCACCCGAGGTCTGTA	GAAC TGAGCCACCCGAGGTCTGTA
		AAAA TACAGACCTCGGGTGGCTCA
Trp53-site15	TAGTGGATGGTGGTATACTC	GAAC TAGTGGATGGTGGTATACTC
		AAAA GAGTATACCACCATCCACTA
Trp53-site16	CACATGTACTTGTAGTGGAT	GAAC CACATGTACTTGTAGTGGAT
		AAAA ATCCACTACAAGTACATGTG
Trp53-site17	CCATCATCACACTGGAAGAC	GAAC CCATCATCACACTGGAAGAC
		AAAA GTCTTCCAGTGTGATGATGG
Trp53-site18	AGGTTCGTGTTTGTGCCTGC	GAAC AGGTTCGTGTTTGTGCCTGC
		AAAA GCAGGCACAAACACGAACCT
Trp53-site19	TGCCTGCCCTGGGAGAGACC	GAAC TGCCTGCCCTGGGAGAGACC
		AAAA GGTCTCTCCCAGGGCAGGCA
Trp53-site20	CGGAAATTTTCTTCTTCTGT	GAAC CGGAAATTTTCTTCTTCTGT
		AAAA ACAGAAGAAGAAAATTTCCG
Trp53-site21	CCCTGAACTGCCCCCAGGGA	GAAC CCCTGAACTGCCCCCAGGGA
		AAAA TCCCTGGGGGCAGTTCAGGG
Trp53-site22	TGCAGGTGGGCAGCGCTGTG	GAAC TGCAGGTGGGCAGCGCTGTG

		AAAA CACAGCGCTGCCCACCTGCA
Trp53-site23	CGGGGGAGAGGCGCTTGTGC	GAAC CGGGGGAGAGGCGCTTGTGC
		AAAA GCACAAGCGCCTCTCCCCCG
Trp53-site24	ATGGAGAGTATTTCACCCTC	GAAC ATGGAGAGTATTTCACCCTC
		AAAA GAGGGTGAAATACTCTCCAT
Trp53-site25	AGGGTGAAATACTCTCCATC	GAAC AGGGTGAAATACTCTCCATC
		AAAA GATGGAGAGTATTTCACCCT
Trp53-site26	GTACCTTGAGGGTGAAATAC	GAAC GTACCTTGAGGGTGAAATAC
		AAAA GTATTTCACCCTCAAGGTAC
Trp53-site27	CGCCCGCGGATCTGCAGCAG	GAAC CGCCCGCGGATCTGCAGCAG
		AAAA CTGCTGCAGATCCGCGGGCG
Trp53-site28	GAGTTAAAGGATGCCCATGC	GAAC GAGTTAAAGGATGCCCATGC
		AAAA GCATGGGCATCCTTTAACTC
Trp53-site29	AAGGATGCCCATGCTACAGA	GAAC AAGGATGCCCATGCTACAGA
		AAAA TCTGTAGCATGGGCATCCTT
Trp53-site30	CCTGGAGTGAGCCCTGCTGT	GAAC CCTGGAGTGAGCCCTGCTGT
		AAAA ACAGCAGGGCTCACTCCAGG

Table S3. SpCas12f1 target site sequences in this study.

sgRNA	Target site sequences (5'-3')	oligos
Trp53-site1	CAGGCTTATGGAAACTGTGA	AAAC CAGGCTTATGGAAACTGTGA
		AAAA TCACAGTTTCCATAAGCCTG
Trp53-site2	CATAAGCCTGAAAATGTCTC	AAAC CATAAGCCTGAAAATGTCTC
		AAAA GAGACATTTTCAGGCTTATG
Trp53-site3	TTGAAGGCCCAAGTGAAGCC	AAAC TTGAAGGCCCAAGTGAAGCC
		AAAA GGCTTCACTTGGGCCTTCAA
Trp53-site4	TGAAGGCCCAAGTGAAGCCC	AAAC TGAAGGCCCAAGTGAAGCCC

		AAAA GGGCTTCACTTGGGCCTTCA
Trp53-site5	GAAGGCCCAAGTGAAGCCCT	AAAC GAAGGCCCAAGTGAAGCCCT
		AAAA AGGGCTTCACTTGGGCCTTC
Trp53-site6	AAGGCCCAAGTGAAGCCCTC	AAAC AAGGCCCAAGTGAAGCCCTC
		AAAA GAGGGCTTCACTTGGGCCTT
Trp53-site7	GTCCCTTCTCAAAAAACTTA	AAAC GTCCCTTCTCAAAAAACTTA
		AAAA TAAGTTTTTTGAGAAGGGAC
Trp53-site8	TCCCTTCTCAAAAAACTTAC	AAAC TCCCTTCTCAAAAAACTTAC
		AAAA GTAAGTTTTTTGAGAAGGGA
Trp53-site9	TCATCTTTTGTCCCTTCTCA	AAAC TGAGAAGGGACAAAAGATGA
		AAAA TCATCTTTTGTCCCTTCTCA
Trp53-site10	GTCATCTTTTGTCCCTTCTC	AAAC GAGAAGGGACAAAAGATGAC
		AAAA GTCATCTTTTGTCCCTTCTC
Trp53-site11	AGAAGGGACAAAAGATGACA	AAAC AGAAGGGACAAAAGATGACA
		AAAA TGTCATCTTTTGTCCCTTCT
Trp53-site12	CTTCCACCCGGATAAGATGC	AAAC CTTCCACCCGGATAAGATGC
		AAAA GCATCTTATCCGGGTGGAAG
Trp53-site13	TATCCCGAGTATCTGGAAGA	AAAC TATCCCGAGTATCTGGAAGA
		AAAA TCTTCCAGATACTCGGGATA
Trp53-site14	CGCCACAGCGTGGTGGTACC	AAAC CGCCACAGCGTGGTGGTACC
		AAAA GGTACCACCACGCTGTGGCG
Trp53-site15	TTCTTCTGTACGGCGGTCTC	AAAC TTCTTCTGTACGGCGGTCTC
		AAAA GAGACCGCCGTACAGAAGAA
Trp53-site16	CTTCTTCTGTACGGCGGTCT	AAAC CTTCTTCTGTACGGCGGTCT
		AAAA AGACCGCCGTACAGAAGAAG
Trp53-site17	CGGAAATTTTCTTCTTCTGT	AAAC CGGAAATTTTCTTCTTCTGT
		AAAA

		ACAGAAGAAGAAAATTTCCG
Trp53-site18	CGCAAAAAGGAAGTCCTTTG	AAAC CGCAAAAAGGAAGTCCTTTG
		AAAA CAAAGGACTTCCTTTTTGCG
Trp53-site19	TGCGGAAATTTTCTTCTTCT	AAAC TGCGGAAATTTTCTTCTTCT
		AAAA AGAAGAAGAAAATTTCCGCA
Trp53-site20	GCGGAAATTTTCTTCTTCTG	AAAC GCGGAAATTTTCTTCTTCTG
		AAAA CAGAAGAAGAAAATTTCCGC
Trp53-site21	CGCTCCCTGGGGGCAGTTCA	AAAC CGCTCCCTGGGGGCAGTTCA
		AAAA TGAACTGCCCCCAGGGAGCG
Trp53-site22	CGGGGGAGAGGCGCTTGTGC	AAAC CGGGGGAGAGGCGCTTGTGC
		AAAA GCACAAGCGCCTCTCCCCCG
Trp53-site23	GCGGGGGAGAGGCGCTTGTG	AAAC GCGGGGGAGAGGCGCTTGTG
		AAAA CACAAGCGCCTCTCCCCCGC
Trp53-site24	TTTTGCGGGGGAGAGGCGCT	AAAC TTTTGCGGGGGAGAGGCGCT
		AAAA AGCGCCTCTCCCCCGCAAAA
Trp53-site25	CTTTTGCGGGGGAGAGGCGC	AAAC CTTTTGCGGGGGAGAGGCGC
		AAAA GCGCCTCTCCCCCGCAAAAG
Trp53-site26	TTCTTTTGCGGGGGAGAGGC	AAAC TTCTTTTGCGGGGGAGAGGC
		AAAA GCCTCTCCCCCGCAAAAGAA
Trp53-site27	ACCCTCAAGGTACCAAGGCT	AAAC ACCCTCAAGGTACCAAGGCT
		AAAA AGCCTTGGTACCTTGAGGGT
Trp53-site28	CGCCCGCGGATCTGCAGCAG	AAAC CGCCCGCGGATCTGCAGCAG
		AAAA CTGCTGCAGATCCGCGGGCG
Trp53-	ACTCTAAGGCCTCATTCAGC	AAAC

site29		ACTCTAAGGCCTCATTCAGC
		AAAA GCTGAATGAGGCCTTAGAGT

Table S4. Un1Cas12f1 target site sequences in this study.

sgRNA	Target site sequences (5’-3’)	oligos
Trp53-site1	CAGGCTTATGGAAACTGTGA	CAAC CAGGCTTATGGAAACTGTGA
		AAAA TCACAGTTTCCATAAGCCTG
Trp53-site2	CATAAGCCTGAAAATGTCTC	CAAC CATAAGCCTGAAAATGTCTC
		AAAA GAGACATTTTCAGGCTTATG
Trp53-site3	TTGAAGGCCCAAGTGAAGCC	CAAC TTGAAGGCCCAAGTGAAGCC
		AAAA GGCTTCACTTGGGCCTTCAA
Trp53-site4	TGAAGGCCCAAGTGAAGCCC	CAAC TGAAGGCCCAAGTGAAGCCC
		AAAA GGGCTTCACTTGGGCCTTCA
Trp53-site5	GAAGGCCCAAGTGAAGCCCT	CAAC GAAGGCCCAAGTGAAGCCCT
		AAAA AGGGCTTCACTTGGGCCTTC
Trp53-site6	AAGGCCCAAGTGAAGCCCTC	CAAC AAGGCCCAAGTGAAGCCCTC
		AAAA GAGGGCTTCACTTGGGCCTT
Trp53-site7	GTCCCTTCTCAAAAAACTTA	CAAC GTCCCTTCTCAAAAAACTTA
		AAAA TAAGTTTTTTGAGAAGGGAC
Trp53-site8	TCCCTTCTCAAAAAACTTAC	CAAC TCCCTTCTCAAAAAACTTAC
		AAAA GTAAGTTTTTTGAGAAGGGA
Trp53-site9	TTGAGAAGGGACAAAAGATG	CAAC TTGAGAAGGGACAAAAGATG
		AAAA CATCTTTTGTCCCTTCTCAA
Trp53-site10	TGAGAAGGGACAAAAGATGA	CAAC TGAGAAGGGACAAAAGATGA
		AAAA TCATCTTTTGTCCCTTCTCA
Trp53-site11	GAGAAGGGACAAAAGATGAC	CAAC GAGAAGGGACAAAAGATGAC
		AAAA GTCATCTTTTGTCCCTTCTC
Trp53-site12	AGAAGGGACAAAAGATGACA	CAAC AGAAGGGACAAAAGATGACA
		AAAA TGTCATCTTTTGTCCCTTCT

Trp53-site13	CTTCCACCCGGATAAGATGC	CAAC CTTCCACCCGGATAAGATGC<br>AAAA GCATCTTATCCGGGTGGAAG
Trp53-site14	TATCCCGAGTATCTGGAAGA	CAAC TATCCCGAGTATCTGGAAGA<br>AAAA TCTTCCAGATACTCGGGATA
Trp53-site15	CGCCACAGCGTGGTGGTACC	CAAC CGCCACAGCGTGGTGGTACC<br>AAAA GGTACCACCACGCTGTGGCG
Trp53-site16	GCCACAGCGTGGTGGTACCT	CAAC GCCACAGCGTGGTGGTACCT<br>AAAA AGGTACCACCACGCTGTGGC
Trp53-site17	AGGTTCGTGTTTGTGCCTGC	CAAC AGGTTCGTGTTTGTGCCTGC<br>AAAA GCAGGCACAAACACGAACCT
Trp53-site18	TTCTTCTGTACGGCGGTCTC	CAAC TTCTTCTGTACGGCGGTCTC<br>AAAA GAGACCGCCGTACAGAAGAA
Trp53-site19	CTTCTTCTGTACGGCGGTCT	CAAC CTTCTTCTGTACGGCGGTCT<br>AAAA AGACCGCCGTACAGAAGAAG
Trp53-site20	CGGAAATTTTCTTCTTCTGT	CAAC CGGAAATTTTCTTCTTCTGT<br>AAAA ACAGAAGAAGAAAATTTCCG
Trp53-site21	CGCAAAAAGGAAGTCCTTTG	CAAC CGCAAAAAGGAAGTCCTTTG<br>AAAA CAAAGGACTTCCTTTTTGCG
Trp53-site22	TGCGGAAATTTTCTTCTTCT	CAAC TGCGGAAATTTTCTTCTTCT<br>AAAA AGAAGAAGAAAATTTCCGCA
Trp53-site23	GCGGAAATTTTCTTCTTCTG	CAAC GCGGAAATTTTCTTCTTCTG<br>AAAA CAGAAGAAGAAAATTTCCGC
Trp53-site24	CCCTGAACTGCCCCCAGGGA	CAAC CCCTGAACTGCCCCCAGGGA<br>AAAA TCCCTGGGGGCAGTTCAGGG

Trp53-site25	CGCTCCCTGGGGGCAGTTCA	CAAC CGCTCCCTGGGGGCAGTTCA
		AAAA TGAACTGCCCCCAGGGAGCG
Trp53-site26	CGGGGGAGAGGCGCTTGTGC	CAAC CGGGGGAGAGGCGCTTGTGC
		AAAA GCACAAGCGCCTCTCCCCCG
Trp53-site27	GCGGGGGAGAGGCGCTTGTG	CAAC GCGGGGGAGAGGCGCTTGTG
		AAAA CACAAGCGCCTCTCCCCCGC
Trp53-site28	TTTTGCGGGGGAGAGGCGCT	CAAC TTTTGCGGGGGAGAGGCGCT
		AAAA AGCGCCTCTCCCCCGCAAAA
Trp53-site29	CTTTTGCGGGGGAGAGGCGC	CAAC CTTTTGCGGGGGAGAGGCGC
		AAAA GCGCCTCTCCCCCGCAAAAG
Trp53-site30	TCTTTTGCGGGGGAGAGGCG	CAAC TCTTTTGCGGGGGAGAGGCG
		AAAA CGCCTCTCCCCCGCAAAAGA
Trp53-site31	TTCTTTTGCGGGGGAGAGGC	CAAC TTCTTTTGCGGGGGAGAGGC
		AAAA GCCTCTCCCCCGCAAAAGAA
Trp53-site32	ACCCTCAAGGTACCAAGGCT	CAAC ACCCTCAAGGTACCAAGGCT
		AAAA AGCCTTGGTACCTTGAGGGT
Tyr-site1	GTATTGCCTTCTGTGGAGTT	CAAC GTATTGCCTTCTGTGGAGTT
		AAAA AACTCCACAGAAGGCAATAC
Tyr-site2	TATTGCCTTCTGTGGAGTTT	CAAC TATTGCCTTCTGTGGAGTTT
		AAAA AAACTCCACAGAAGGCAATA
Tyr-site3	CAGATCTCTGATGGCCATTT	CAAC CAGATCTCTGATGGCCATTT
		AAAA AAATGGCCATCAGAGATCTG
Tyr-site4	CCTCGAGCCTGTGCCTCCTC	CAAC CCTCGAGCCTGTGCCTCCTC

		AAAA GAGGAGGCACAGGCTCGAGG
Tyr-site5	CTCGAGCCTGTGCCTCCTCT	CAAC CTCGAGCCTGTGCCTCCTCT
		AAAA AGAGGAGGCACAGGCTCGAG
Tyr-site6	GCCAACAAGTTCTTAGAGGA	CAAC GCCAACAAGTTCTTAGAGGA
		AAAA TCCTCTAAGAACTTGTTGGC
Tyr-site7	AGGCAGAGGTTCCTGCCAGG	CAAC AGGCAGAGGTTCCTGCCAGG
		AAAA CCTGGCAGGAACCTCTGCCT
Tyr-site8	AAGGGGAACTGAGGTCCAGA	CAAC AAGGGGAACTGAGGTCCAGA
		AAAA TCTGGACCTCAGTTCCCCTT
Tyr-site9	ATAATAGGACCTGCCAGTGC	CAAC ATAATAGGACCTGCCAGTGC
		AAAA GCACTGGCAGGTCCTATTAT
Tyr-site10	TAATAGGACCTGCCAGTGCT	CAAC TAATAGGACCTGCCAGTGCT
		AAAA AGCACTGGCAGGTCCTATTA
Tyr-site11	AACTGCGGAAACTGTAAGTT	CAAC AACTGCGGAAACTGTAAGTT
		AAAA AACTTACAGTTTCCGCAGTT
Tyr-site12	CGCAGTTGAAACCCATGAAG	CAAC CGCAGTTGAAACCCATGAAG
		AAAA CTTCATGGGTTTCAACTGCG
Tyr-site13	GATTTGGGGGCCCAAATTGT	CAAC GATTTGGGGGCCCAAATTGT
		AAAA ACAATTTGGGCCCCCAAATC
Tyr-site14	GGGGCCCAAATTGTACAGAG	CAAC GGGGCCCAAATTGTACAGAG
		AAAA CTCTGTACAATTTGGGCCCC
Tyr-site15	GGCCCCCAAATCCAAACTTA	CAAC GGCCCCCAAATCCAAACTTA
		AAAA TAAGTTTGGATTTGGGGGCC
Tyr-site16	TTCTAATCAAGACTCGCTTC	CAAC TTCTAATCAAGACTCGCTTC

		AAAA GAAGCGAGTCTTGATTAGAA
Tyr-site17	GATTTGAGTGTCTCCGAAAA	CAAC GATTTGAGTGTCTCCGAAAA
		AAAA TTTTCGGAGACACTCAAATC
Tyr-site18	ATTTGAGTGTCTCCGAAAAG	CAAC ATTTGAGTGTCTCCGAAAAG
		AAAA CTTTTCGGAGACACTCAAAT
Tyr-site19	GGAGACACTCAAATCAAAAA	CAAC GGAGACACTCAAATCAAAAA
		AAAA TTTTTGATTTGAGTGTCTCC
Tyr-site20	CGGAGACACTCAAATCAAAA	CAAC CGGAGACACTCAAATCAAAA
		AAAA TTTTGCTAAAGTGAGGTAAG
Tyr-site21	TTACCTCACTTTAGCAAAAC	CAAC TTACCTCACTTTAGCAAAAC
		AAAA GTTTTGCTAAAGTGAGGTAA
Tyr-site22	GCAAAACATACTATCAGCTC	CAAC GCAAAACATACTATCAGCTC
		AAAA GAGCTGATAGTATGTTTTGC
Tyr-site23	CTAAAGTGAGGTAAGAAAAG	CAAC CTAAAGTGAGGTAAGAAAAG
		AAAA CTTTTCTTACCTCACTTTAG
Tyr-site24	GCTAAAGTGAGGTAAGAAAA	CAAC GCTAAAGTGAGGTAAGAAAA
		AAAA TTTTCTTACCTCACTTTAGC
Tyr-site25	GCCATAGGTGCCTGTGGGGA	CAAC GCCATAGGTGCCTGTGGGGA
		AAAA TCCCCACAGGCACCTATGGC
Tyr-site26	ATGATATCAACATCTACGAC	CAAC ATGATATCAACATCTACGAC
		AAAA GTCGTAGATGTTGATATCAT
Tyr-site27	TATGGATGCATTACTATGTG	CAAC TATGGATGCATTACTATGTG
		AAAA CACATAGTAATGCATCCATA
Tyr-site28	AGAGCCCCCAAGCAGTGTGT	CAAC AGAGCCCCCAAGCAGTGTGT
		AAAA ACACACTGCTTGGGGGCTCT
Tyr-site29	GCCCATGAAGCACCAGGGTT	CAAC GCCCATGAAGCACCAGGGTT
		AAAA AACCCTGGTGCTTCATGGGC
Tyr-site30	CCCATGAAGCACCAGGGTTT	CAAC CCCATGAAGCACCAGGGTTT
		AAAA

		AAACCCTGGTGCTTCATGGG
Tyr-site31	TGCCTTGGCACAGACTTTTC	CAAC TGCCTTGGCACAGACTTTTC
		AAAA GAAAAGTCTGTGCCAAGGCA
Tyr-site32	CTTGTTATTGTGGGAACAAG	CAAC CTTGTTATTGTGGGAACAAG
		AAAA CTTGTTCCCACAATAACAAG
Tyr-site33	TTGTTATTGTGGGAACAAGA	CAAC TTGTTATTGTGGGAACAAGA
		AAAA TCTTGTTCCCACAATAACAA
Tyr-site34	TTGTTCCCACAATAACAAGA	CAAC TTGTTCCCACAATAACAAGA
		AAAA TCTTGTTATTGTGGGAACAA
Tyr-site35	TGCATCTCTCCAATCCCAGT	CAAC TGCATCTCTCCAATCCCAGT
		AAAA ACTGGGATTGGAGAGATGCA
Tyr-site36	CTGCATCTCTCCAATCCCAG	CAAC CTGCATCTCTCCAATCCCAG
		AAAA CTGGGATTGGAGAGATGCAG
Tyr-site37	CACAGATGAGTACTTGGGAG	CAAC CACAGATGAGTACTTGGGAG
		AAAA CTCCCAAGTACTCATCTGTG
Tyr-site38	AGGGTGACGACCTCCCAAGT	CAAC AGGGTGACGACCTCCCAAGT
		AAAA ACTTGGGAGGTCGTCACCCT
Tyr-site39	CAGGGTGACGACCTCCCAAG	CAAC CAGGGTGACGACCTCCCAAG
		AAAA CTTGGGAGGTCGTCACCCTG
Hpd-site1	CACTCGGTGACCTTCTGGGT	CAAC CACTCGGTGACCTTCTGGGT
		AAAA ACCCAGAAGGTCACCGAGTG
Hpd-site2	CAGGCTGCTTCCTTCTACTG	CAAC CAGGCTGCTTCCTTCTACTG
		AAAA CAGTAGAAGGAAGCAGCCTG
Hpd-site3	AACCTCTGGCCTACAGGGGC	CAAC AACCTCTGGCCTACAGGGGC
		AAAA

		GCCCCTGTAGGCCAGAGGTT
Hpd-site4	CCTTGCTTGATGACGTGGCT	CAAC CCTTGCTTGATGACGTGGCT
		AAAA AGCCACGTCATCAAGCAAGG
Hpd-site5	GATTGTGTTTGTTCTCTGCT	CAAC GATTGTGTTTGTTCTCTGCT
		AAAA AGCAGAGAACAAACACAATC
Hpd-site6	TTCTCTGCTCTGCTCTCAAT	CAAC TTCTCTGCTCTGCTCTCAAT
		AAAA ATTGAGAGCAGAGCAGAGAA
Hpd-site7	TTCCAGGGATTGAGAGCAGA	CAAC TTCCAGGGATTGAGAGCAGA
		AAAA TCTGCTCTCAATCCCTGGAA
Hpd-site8	ACCCCGTCGCCATGCTTCAC	CAAC ACCCCGTCGCCATGCTTCAC
		AAAA GTGAAGCATGGCGACGGGGT
Hpd-site9	GCGCCCCGTTCTCGAGCTTT	CAAC GCGCCCCGTTCTCGAGCTTT
		AAAA AAAGCTCGAGAACGGGGCGC
Hpd-site10	TCTTGCTCCACCCATGGCTC	CAAC TCTTGCTCCACCCATGGCTC
		AAAA GAGCCATGGGTGGAGCAAGA
Hpd-site11	GGAAGGTGAAGTTTGCTGTG	CAAC GGAAGGTGAAGTTTGCTGTG
		AAAA CACAGCAAACTTCACCTTCC
Hpd-site12	CTGTGCTGCAGACGGTGAGT	CAAC CTGTGCTGCAGACGGTGAGT
		AAAA ACTCACCGTCTGCAGCACAG
Hpd-site13	TTACCTGGATTCGAGGCCCC	CAAC TTACCTGGATTCGAGGCCCC
		AAAA GGGGCCTCGAATCCAGGTAA
Hpd-site14	GAAGCAGGGTATCCTTGTAT	CAAC GAAGCAGGGTATCCTTGTAT
		AAAA ATACAAGGATACCCTGCTTC
Hpd-site15	TTGGTCGGGTTGGTTGCCTA	CAAC TTGGTCGGGTTGGTTGCCTA

		AAAA TAGGCAACCAACCCGACCAA
Hpd-site16	AGGTACCTGCAGAGTGGCCA	CAAC AGGTACCTGCAGAGTGGCCA
		AAAA TGGCCACTCTGCAGGTACCT
Hpd-site17	ATGGATTCCTCGTAGTTGGT	CAAC ATGGATTCCTCGTAGTTGGT
		AAAA ACCAACTACGAGGAATCCAT
Hpd-site18	GATGGATTCCTCGTAGTTGG	CAAC GATGGATTCCTCGTAGTTGG
		AAAA CCAACTACGAGGAATCCATC
Hpd-site19	TAGTAAGAAGATGGGGCGGC	CAAC TAGTAAGAAGATGGGGCGGC
		AAAA GCCGCCCCATCTTCTTACTA
Hpd-site20	TCGTCATAGTCGACTAGGAT	CAAC TCGTCATAGTCGACTAGGAT
		AAAA ATCCTAGTCGACTATGACGA
Hpd-site21	GAGCGGGCAACTTCAACTCT	CAAC GAGCGGGCAACTTCAACTCT
		AAAA AGAGTTGAAGTTGCCCGCTC
Hpd-site22	AGATTGTGTTTGTTCTCTGC	CAAC AGATTGTGTTTGTTCTCTGC
		AAAA GCAGAGAACAAACACAATCT
Hpd-site23	TAGATTGTGTTTGTTCTCTG	CAAC TAGATTGTGTTTGTTCTCTG
		AAAA CAGAGAACAAACACAATCTA
Hpd-site24	GGCGCCCCGTTCTCGAGCTT	CAAC GGCGCCCCGTTCTCGAGCTT
		AAAA AAGCTCGAGAACGGGGCGCC

Table S5. CjCas9 target site sequences in this study.

sgRNA	Target site sequences (5'-3')	Primer sequences (5'-3')
Hpd-site1	GGGATTGAGAGCAGAGCAGAGA	CACCGGGGATTGAGAGCAGAGCAGAGA
		AAAC TCTCTGCTCTGCTCTCAATCCCC
Hpd-site2	TTTGTCTTGCTCCACCCATGGC	CACC G TTTGTCTTGCTCCACCCATGGC
		AAACGCCATGGGTGGAGCAAGACAAAC
Hpd-site3	GCGTCAGTGTTCACTCACCGTC	CACC G GCGTCAGTGTTCACTCACCGTC

		AAACGACGGTGAGTGAACACTGACGC C
Hpd-site4	CTTCTCACCCCCAGTATGGAG A	CACC G CTTCTCACCCCCAGTATGGAGA
		AAAC TCTCCATACTGGGGGTGAGAAG C
Hpd-site5	TCACCCCCAGTATGGAGATAC C	CACC G TCACCCCCAGTATGGAGATACC
		AAAC GGTATCTCCATACTGGGGGTGA C
Hpd-site6	TCCACCAGGGTGTGTGTGGTA T	CACC G TCCACCAGGGTGTGTGTGGTAT
		AAAC ATACCACACACACCCTGGTGGA C
Hpd-site7	ACACACCCTGGTGGAGAAGAT C	CACCG ACACACCCTGGTGGAGAAGATC
		AAAC GATCTTCTCCACCAGGGTGTGT C
Hpd-site8	TTCTTACCTGGATTCGAGGCC C	CACC G TTCTTACCTGGATTCGAGGCCC
		AAACGGGCCTCGAATCCAGGTAAGAA C
Hpd-site9	TGGATTCGAGGCCCCAACATA C	CACC G TGGATTCGAGGCCCCAACATAC
		AAAC GTATGTTGGGGCCTCGAATCCA C
Hpd-site10	GTTCCACCGGTTCTGGTCCGT G	CACC G GTTCCACCGGTTCTGGTCCGTG
		AAACCACGGACCAGAACCGGTGGAA CC
Hpd-site11	TTCTGGTCCGTGGACGACACG C	CACC G TTCTGGTCCGTGGACGACACGC
		AAACGCGTGTCGTCCACGGACCAGAA C
Hpd-site12	CTGGTCCGTGGACGACACGC AG	CACCG CTGGTCCGTGGACGACACGCAG
		AAAC CTGCGTGTCGTCCACGGACCAG C
Hpd-site13	GTGGACGACACGCAGGTGCA CA	CACCGGTGGACGACACGCAGGTGCA CA
		AAAC TGTGCACCTGCGTGTCGTCCAC C
Hpd-site14	GGAGCGCAGAGAGCTGTACTC C	CACCG GGAGCGCAGAGAGCTGTACTCC

		AAAC GGAGTACAGCTCTCTGCGCTCC C
Hpd-site15	GACTATAATGGGGGTGCTGGG G	CACC G GACTATAATGGGGGTGCTGGGG
		AAAC CCCCAGCACCCCCATTATAGTC C
Hpd-site16	TCTTGAGAGCGATGTGCTGGA C	CACC G TCTTGAGAGCGATGTGCTGGAC
		AAAC GTCCAGCACATCGCTCTCAAGA C
Hpd-site17	ACAGATCCGCCACTTGAGGGA G	CACC G ACAGATCCGCCACTTGAGGGAG
		AAAC CTCCCTCAAGTGGCGGATCTGT C
Hpd-site18	TCACCAAGCCCATGCAGGACC G	CACC G TCACCAAGCCCATGCAGGACCG
		AAAC CGGTCCTGCATGGGCTTGGTGA C
Hpd-site19	AAGTCATTCAACGTCACAACCA	CACC G AAGTCATTCAACGTCACAACCA
		AAAC TGGTTGTGACGTTGAATGACTT C

Table S6. PCR and IVT primers used in this study.

Primer	Primer sequences (5'-3')
mTrp53-exon1-1f	TATTCTACCCTTTCCTATAAGCC
mTrp53-exon1-1r	AGGCTGGCCTCCAACTTGCTATG
mTrp53-exon1-2f	CATACAGTACACAATCTCTT
mTrp53-exon1-2r	GATTTACAGACACCCAACAC
mTrp53-exon3-1f	CAGAAGATATCCTGGTAAGGCCC
mTrp53-exon3-1r	TGGAGAGATGCAGAGAATATGAG
mTrp53-exon3-2f	CTTTGGTGTTGGGCTGGTAG
mTrp53-exon3-2r	GGCAAAACTAAACTCTGAGG
mTrp53-exon4-1f	AGGTCCCAGTCCTCTCTTTGCTG
mTrp53-exon4-1r	TTAAAGTAGACCCTGGGCTGGGC
mTrp53-exon4-2f	ATGGTGCTTGGACAATGTGT
mTrp53-exon4-2r	TAAGCCTAGCTAGCACTCAG
mTrp53-exon6-1f	ATCCCTACTCTACAACTAAAACT
mTrp53-exon6-1r	AAGGTCCAGTTACAGGAACCCCG
mTrp53-exon6-2f	ATTAGAGGCTATAGCCAGCC
mTrp53-exon6-2r	GACTTCATTTAGGTAGATAG
mTrp53-exon7-1f	CCTTTGGCTGCAGATATGACAAG
mTrp53-exon7-1r	GCACAGCCTCAGAGCATGAGCTC

mTrp53-exon7-2f	CTAGTTTACACACAGTCAGG
mTrp53-exon7-2r	AGATAAAGCCACTGAAAAAG
mTrp53-exon9-1f	TGGAGCCAGCTTAAGTTGGGAAC
mTrp53-exon9-1r	TGCAGCCCTAAGCATCTAGCAGG
mTrp53-exon9-2f	TAAAATCGTGAAAGTGGTTG
mTrp53-exon9-2r	TACAAAGGCTGAGCTGGAGG
mHpd-exon2-1f	GAGCAGGGTGAGTCCCATTCTCG
mHpd-exon2-1r	CAAGGTTCCAAAGTGCCAGTCCT
mHpd-exon2-2f	CTGCTTCCTGGGACTCATCC
mHpd-exon2-2r	CACTAGCCAATCCCAGTTCC
mHpd-exon3-1f	CAATCAGGGTCCCCAAGGACCTT
mHpd-exon3-1r	GAGAAGTTTGAAACCAGGAAGAT
mHpd-exon3-2f	AGAGTCTCCAAATGACGGAC
mHpd-exon3-2r	TACATCTTGGAACCAGCTAG
mHpd-exon4-1f	CTAGAACACGTGGTCCAGGGATC
mHpd-exon4-1r	GGCATTAGCTATCCCCATTTTGC
mHpd-exon4-2f	CAGAGGTGCCATAGAGCATG
mHpd-exon4-2r	CAGGGCAGACACACAGAAAG
mHpd-Exon6-1f	CCAGGTGACAAAGGATCCAGCTG
mHpd-Exon6-1r	GTAACTCCCAGAAGCCTCTGTGT
mHpd-Exon6-2f	CTTATATTAGGACTACCCAG
mHpd-Exon6-2r	CATTCTCAAAGGTCAGGCTT
mHpd-exon7-1f	CTGAGTTAGGGTCAGCTTCATGG
mHpd-exon7-1r	AAATGACGGAGCTGCCTGTGAAC
mHpd-exon7-2f	TAGAGAAGAGTGGGGGCTTT
mHpd-exon7-2r	GTTTCCCACCAGATGCTTAC
mHpd-exon8-1f	CATCAGGGCCTAATACGCCATCT
mHpd-exon8-1r	GTTCTCTGGCCTCCCAACATGTG
mHpd-exon8-2f	TATAGGTAAAGGGTTCATGG
mHpd-exon8-2r	CATATAATAAGCACACAGCC
mHpd-exon9-1f	TGAGGATCCTGTGTAACGGGTGT
mHpd-exon9-1r	GTTTGTGGGAGAGGAAAGGGACG
mHpd-exon9-2f	GAAGAGGGTGGGAAGGTCTC
mHpd-exon9-2r	CGCTACTCTCATCGGCAGAG
mHpd-exon10-1f	CTATAAGTGAGAGCCATCACTAG
mHpd-exon10-1r	TCTCCTATAGATTTCAGTTAAAG
mHpd-exon10-2f	TATCCTGACAGAACGTGGAT
mHpd-exon10-2r	TCAAGTGGTCTGCTGTAACT
mHpd-exon11-1f	ACACTTTCCAAGGCTCCCACAAG
mHpd-exon11-1r	GGCAAGGCGGACTCTTGTAATAG
mHpd-exon11-2f	CAAGGTCCAGTTTACCTTCC
mHpd-exon11-2r	TGGGTGGAGCCCTGAGGTTC
mTyr-1f	AACAGGCTGAGAGTATTTGATGT

mTyr-1r	ATTTTCTGCCCTGAGATATTATC
mTyr-2f	GTTGCTGGAAAAGAAGTCTG
mTyr-2r	TGCTGAAGTACCAGTCTTTG
Un1Cas12f1-Tyr-1f	GAATTC NNNNNN GGATCC TAAGTTTGGATTTGGGGGCC
Un1Cas12f1-Tyr-1r	TGATATCATTAAACATGGGT
Un1Cas12f1-Tyr-2f	GAATTC NNNNNN GGATCC CAGAGAAGCGAGTCTTGATT
Un1Cas12f1-Tyr-2r	GCATCCATACAAAGAGGTCG
Un1Cas12f1-Fah-1f	GAATTC NNNNNN GGATCC CTACTTGGTACCCCACGCAG
Un1Cas12f1-Fah-1r	GGACTCTGAGGTATGCAAAT
CjCas9-Fah-1f	GAATTC NNNNNN GGATCC TAGAGCATGCCAGGAAGATG
CjCas9-Fah-1r	TCACCCTTGCCCCAACCTAC
Un1Cas12f1-Hpd-site12-1f	GAAATTAATACGACTCACTATAGG G ACCGCTTCACTTAGAGTGA
Un1Cas12f1-Hpd-site12-1r	CTCACCGTCTGCAGCACAG GTTGCATTCCTTTCTTTGT
Un1Cas12f1-Tyr-site18-1r	CTTTTCGGAGACACTCAAAT GTTGCATTCCTTTCTTTGT
Un1Cas12f1-Tyr-site25-1r	TCCCCACAGGCACCTATGGC GTTGCATTCCTTTCTTTGT

Table S7. Potential off-target sites of Hpd in the mouse genome.

No.	sequences (5’-3’)	Primer sequences (5’-3’)
Un1Cas12f1-Hpd-OT1	TTTG CTaTGCTGCAGAtGGcaAGT	1f: CTGGGCACAGTTCATACTTCAG<br>1r: TCCTCCTTCTCCTTATCCTCCT
		2f: CTAGAAACCGCTGGATCAAAG<br>2r: TCTGCCTCCTGAGTGCTAGATT
Un1Cas12f1-Hpd-OT2	TTTG gTGTGCccCAGAtGGTGAGT	1f: ACTGTTAGGGAAGAGGAAGACG<br>1r: CATTAACTTCCCCAAGTGATGG
		2f: AGTCCCCGTGAGGATGAGAT<br>2r: GACTTAAGGCTTTGGGAGATCA
Un1Cas12f1-Hpd-OT3	TTTG CTGTGCTGCAcACccTGgGT	1f: CCTCACCCTTCATGTTTATCCT<br>1r: CTTTGAAAAGAGGTCCTTTTGG
		2f: TGACATCCATACGTTTGTTCATC<br>2r: AAGCCTATCTGGACACAAGATCA
Un1Cas12f1-Hpd-OT4	TTTG CTGTGtTGgAtACaGTGAGT	1f: GCACCTGGTTCCTCTGTAAACT<br>1r: AAAATGACTGTCCCCAAAGGTA
		2f: ATGCTGTTGGCCATGTTGATAC<br>2r: CAAAACCCACTGATAGGAAAGC
Un1Cas12f1	TTTG	1f: TTTTTCCCCCTAGTTTCACTCC

-Hpd-OT5	CTGTGCTGtgtACtGTGAG T	1r: CACTCAAAGGAAGCTTCACAAA<br>2f: GCGCGCTTAATCTAATTTCTTC<br>2r: TCCAGGAGATTTGGAAATCAGT
Un1Cas12f1 -Hpd-OT6	TTTG CTGTGgTGgAGAtGGTGtG T	1f: AGCCTCTTTGCTCATTCATAGG<br>1r: AGACAGGGAATGCCACATTAAG<br>2f: CCTCCCCCACTACAGACTACAT<br>2r: GGGTCTGGTATAGGTTTTTGAGA
Un1Cas12f1 -Hpd-OT7	TTTA CTGTGaTGCAGACttgGAG T	1f: TGGGGACAGGAAACATCTACTT<br>1r: GATTCCTCCCGGTAAGTTTTTC<br>2f: TTGGTGTTAGAGATGGGGAACT<br>2r: CCAGTAAGTACCCACAATGAAGC
Un1Cas12f1 -Hpd-OT8	TTTG CTtTcCTGCAGACGGgGAa T	1f: GATTAGCAACTTTACACCCAGGA<br>1r: TGTCTTGTTGGTGATGTGTCTG<br>2f: CAGTGAATTTTGCAAAGTGAGG<br>2r: TGTAATGCCATCAGTATGTGCT
Un1Cas12f1 -Hpd-OT9	TTTG CTtTGCTGCAaACaGTGA Ga	1f: CATGGGGAAGCCTATCTTACAG<br>1r: CAGTTTGGGGATGGAGTCTTTA<br>2f: AACCCATTCAATTTGTACAGCA<br>2r: TAGGGAAAGAATGGAAGATGGA
Un1Cas12f1 -Hpd-OT10	TTTA CTGaGtTGCAGACtGTGtG T	1f: GATAGAGGAAGAAGAGTGTGTGT G<br>1r: AGCCTGTGAAGGCTCACATCTA<br>2f: TTCGAATACAGCTTATACCATTGC<br>2r: CTGGTCTCTCCTCTTTGGTGAT
CjCas9-Hpd-OT1	GGagagGAGAGCAGAGCA GAGATATAATAC	1f: AACCAAGATAGGGATGCTTTGC<br>1r: CAGCCACACAGATAGATAGTCAC A<br>2f: AAGGAAGTTAGGCATGGTGTAC<br>2r: TATAGACACAGACACACATGCA
CjCas9-Hpd-OT2	GGGATTtcGAGCAGgcCA GAGAGCTGGTAC	1f: TTCCATATTTTTGCCATTTGGT<br>1r: AGCCTTCTGTGAATAACAACTGA<br>2f: TGTTTGTTTCTAGGTTTTTGACCA<br>2r: AGGACCCCAAGTTTGAAGCTA
CjCas9-Hpd-OT3	GGGAcTGAGAGCtGAGCt GAcAAAGGATAC	1f: TCACATTCTGTCTAGAGCTTGG<br>1r: GATGACGAGGTTGTTCAGTGG<br>2f: CAACTGGGAGCCTTGAAAATAC<br>2r: TGAGGCAGGAGGATAGCATAGT
CjCas9-Hpd-OT4	GGcAgTGAGAGCAaAGaA GAGAGAAGACAC	1f: TGCACACAAGATAGAGGCAACT<br>1r: CAGGCCAATAGGGAATTTCTAA<br>2f: CCAGTCATGTGTGTCAAGTGTC<br>2r: AGTCTGACTGTCTCCCCACTGT
CjCas9-	GGGATTagGAGCAGAGgA	1f: ACAAGAATATGGCCCACAGAAT

Hpd-OT5	GgGAAGGAATAC	1r: TGTCTGCACAATTACAACAGAAA
		2f: CATAGGCCTGAACTGGATTCTC<br>2r: CCTTCATGTTCCTATCCCAGAC
CjCas9-<br>Hpd-OT6	GGGgaTGAGAGgcGAGCA<br>GAGACCAGGCAC	1f: GGAAACAAAAGCCTGTACCTTG<br>1r: GGGACAGTGTCTTCTCCCATAC
		2f: AAAGCTGCAGATGGAACCAC<br>2r: TTCCTGAAGGTCATGTCCATTTT
CjCas9-<br>Hpd-OT7	GtGAcTGttAGCAGAGCAG<br>AGATTCAGTAC	1f: TACAAGAGGGCCACTCACCTAT<br>1r: GGGGACAGGGTCTCACTATGTA
		2f: GGAAGGCAGCTGTTAGAACCTA<br>2r: GTCTCTGCCTCCTGAGTGCT
CjCas9-<br>Hpd-OT8	GGGgTTGAGgGtAaAGCA<br>GAGAGAACACAC	1f: CAGAGAACCAGGCTGAAACACT<br>1r: GATGGTCATATAGTTGCGGAAA
		2f: CTGTCACCCACAGTAAGAGCAC<br>2r: TGCTCTGAATGAGTGGCATATC
CjCas9-<br>Hpd-OT9	GGGAaaGAGAGCAGAGC<br>catGATGGAACAC	1f: TAGGAAGGTAGAGAGCCACTGC<br>1r: ATCAGAGAGGCATAGCAGGAAG
		2f: AGATGCAAGTCACCTGAGAAGC<br>2r: AGAGTCTTCCTCCCTGGATTTT
CjCas9-<br>Hpd-OT10	GGGATTGAaAtCAGgtCAtA<br>GACTTGGCAC	1f: ATAAAACAGCACCAGCCCTAAA<br>1r: CTTTAGTCAAGGAAAAACCAGCA
		2f: CCTACAGAACAAAACCCAGCTC<br>2r: GGCCCAACATAATCAAGTTGTAA
